# Measurement of Shank Length in Live Chickens Using Visible–Infrared Image Fusion and Keypoint Prediction

**DOI:** 10.3390/ani16162624

**Published:** 2026-08-21

**Authors:** Chuang Ma, Rui Chen, Kaixiang Huang, Xueming Yin, Zhaorui Cai, Haowen He, Jianbin Huang, Jikang Yang, Cheng Fang

**Affiliations:** 1 College of Engineering, South China Agricultural University, Guangzhou 510642, China; macscau@gmail.com (C.M.); ruichen0512@stu.scau.edu.cn (R.C.);; 2 State Key Laboratory of Swine and Poultry Breeding Industry, South China Agricultural University, Guangzhou 510642, China

**Keywords:** chicken phenotyping, image fusion, infrared thermography, keypoint prediction, vision-based measurement, YOLOv8s-Pose

## Abstract

Accurate shank-length phenotyping of live chickens is hindered by feather occlusion and uncertain endpoint localization in visible images. We developed a two-stage method that fuses registered visible and infrared images before predicting the two measurement endpoints. The fusion network used a U-Net encoder–decoder with residual blocks, Coordinate Attention, and Strip Pooling, trained with a YUV-guided loss. A YOLOv8s-Pose model with Coordinate Attention and a length-related loss then localized the endpoints. The dataset comprised 100 chickens and 1000 paired visible–infrared acquisitions, separated at the individual level into training, validation, and test sets. On the test set, at the chicken level, the reported mean signed difference, mean absolute error, and root mean square error were 0.129 mm, 0.790 mm, and 0.984 mm, respectively. The Pearson correlation coefficient between model-derived and manual reference measurements was 0.992. Compared with either single-modality input, the fused images reduced the mean absolute error and root mean square error. These findings show that complementary texture and thermal-boundary information can support accurate vision-based shank-length measurement under controlled acquisition conditions.

## Simple Summary

Shank length is used to evaluate skeletal development and growth in chickens, but manual measurement requires restraint and direct contact. We developed a vision-based method that combines visible and infrared images. Visible images provide surface texture, whereas infrared images make the feather–shank boundary easier to distinguish. A fusion network combined these cues, and a keypoint model located the two endpoints used to calculate shank length. In the test set, measurements from the proposed method differed from manual measurements by 0.790 mm on average, with a root mean square error of 0.984 mm and a Pearson correlation coefficient of 0.992. Under the controlled acquisition conditions used here, the method avoided direct caliper contact with the shank and provided accurate automated measurements. Further validation is needed for freely moving chickens and commercial-house conditions.

## 1. Introduction

Poultry farming is an important part of livestock and poultry production. The accurate acquisition of body size phenotypic data from live chickens is important for growth monitoring and breeding management [1,2]. Among the many phenotypic parameters of poultry, shank length is a key biological indicator [3]. Chicken shank length not only serves as a key indicator of skeletal development and health status in live chickens but also has high heritability and is therefore an important criterion for selecting desirable genetic traits in poultry breeding [4]. Accurate shank length data are essential for monitoring growth uniformity within a flock, evaluating feed conversion efficiency, and providing early warnings of disease [5].

Traditional methods for measuring chicken shank length mainly rely on manual operation. Contact measurement is performed using a vernier caliper or measuring tape and usually requires the cooperation of two to three workers, making the process labor-intensive and time-consuming. More importantly, frequent catching and physical contact may cause stress in live chickens, harm animal welfare, and affect their subsequent growth. With the development of precision livestock farming, computer vision-based non-contact body size measurement has received increasing research attention. Image enhancement combined with optimized YOLOv8 models has also been used to improve visual recognition under complex imaging conditions [6]. Using image processing and deep learning algorithms, researchers have made substantial progress in estimating the body dimensions and body weight of large livestock, such as cattle and pigs [7,8,9]. Compared with large livestock such as cattle and pigs, poultry are smaller and have slender legs. In addition, the proximal shank region is easily affected by feathers and changes in posture. Therefore, the non-contact measurement of chicken shank length remains challenging. For automated chicken shank length measurement, Sun et al. [10] used computed tomography (CT) imaging to scan chicken legs and reconstructed the scans as a sequence of slices. An improved YOLOv8 model was then used to identify the anatomical structure of the tibia. Chicken shank length was calculated based on the difference in slice indices and the in-plane coordinate distance. However, CT imaging relies on specialized equipment, involves a relatively complex acquisition process, and presents an issue of ionizing radiation exposure. It is more suitable for the detailed examination of skeletal structures but cannot easily meet the needs of large-scale live phenotypic measurement in terms of on-site deployment, continuous data acquisition, and measurement safety. In contrast, measurement methods based on conventional images are easier to deploy on site. Zheng et al. [1] captured images of chicken legs from front and side views. They used YOLOv5s to identify and extract the chicken claw and shank root regions. They then located the top and bottom keypoints for shank length measurement and calculated the Euclidean distance between the two keypoints to obtain the shank length. This study provided an effective approach for the non-contact measurement of shank length in live chickens. Although methods based on visible images have achieved some success, the chicken shank root is often affected by surrounding feathers under practical production conditions. In visible images, the contour of the proximal shank region is often unclear. As a result, computer vision algorithms have difficulty accurately distinguishing the shank root region. This makes the proximal keypoint for shank length measurement more difficult to locate.

Infrared thermal imaging has received increasing attention because of its sensitivity to temperature distribution. Chicken legs lack the thermal insulation provided by feathers, so their surface temperature is significantly lower than that of the feather-covered trunk. This temperature difference creates strong contrast in infrared images and clearly outlines the legs, helping overcome the limitation of visible images in dealing with feather occlusion. Ansarimovahed et al. [11] used this characteristic to effectively separate the heads and legs of chickens from their bodies. Ma et al. [12] further investigated the fusion of visible and infrared images. Taking advantage of the contour information provided by infrared images and the texture information provided by visible images, they used an improved residual network (ResNet) for shank length regression. The Pearson correlation coefficient (R) obtained using the fusion images reached 0.996.

Although Ma et al. [12] improved the correlation and stability of live chicken shank length measurement by fusing visible and infrared images, their evaluation metrics and measurement approach still have room for improvement. On the one hand, their study mainly used R, the coefficient of variation (CV), floating error, and standard deviation (SD) to evaluate the measurement results. However, it did not report accuracy metrics that directly reflect prediction errors, such as mean absolute error (MAE), root mean square error (RMSE), and systematic bias (Bias). On the other hand, their fusion process was mainly based on the decomposition and reconstruction of global or local wavelet coefficients. This process could effectively integrate texture information from visible images and contour information from infrared images. However, it did not perform end-to-end feature learning specifically for the task of shank length endpoint localization. The representation of task-related information, including the shank root boundary, texture details, and thermal boundaries, therefore still had room for improvement. In addition, Ma et al. [12] directly regressed the shank length value by feeding the fusion images into an improved ResNet model. The model output did not include the two measurement endpoints, the top keypoint (TKP) and bottom keypoint (BKP), which limited its interpretability. When the shank root was obscured by feathers, or when the texture information in the visible images and the thermal boundaries in the infrared images were unclear, small deviations near the endpoints could still affect the final measurement results.

Attention mechanisms are commonly used to guide models to focus on key regions or features [13,14]. For example, Du et al. [15] proposed an adaptive channel-spatial attention mechanism called SCBAM. They introduced a self-attention mechanism into the convolutional block attention module (CBAM) to overcome the limitation of convolution kernels, expand the model’s receptive field, and improve its feature representation ability. Li et al. [16] designed a dual-stream cross-modality fusion network. The network used self-attention blocks to extract modality-specific features and cross-attention blocks to enable feature interaction between panchromatic (PAN) and multispectral (MS) images, significantly improving spectral fidelity and spatial details. Chen et al. [17] introduced multihead attention into a bilayer feature-fusion framework to model interactions among different data modalities. Ma et al. [18] embedded the Squeeze-and-Excitation (SE) channel attention mechanism into the feed-forward neural network of a Transformer. This effectively improved the model’s ability to capture local features. Yan et al. [19] proposed a differential selection module that uses a channel attention weighting mechanism. The module redistributes feature channel weights through normalization to select complementary features across modalities, avoid introducing redundant features, and increase attention to the unique features of each modality. Unlike the studies described above, this study proposes a shank length measurement method based on fusion images and keypoint prediction, with the development of a visible and infrared image fusion model as its core. The method is designed to address unclear shank root boundaries, difficulty in identifying endpoint regions, and insufficient information from a single modality in live chicken shank length measurement. The main work conducted in this study is as follows:

1. We developed U-Net-CA-SPM, a visible–infrared fusion network that combines residual encoding, Coordinate Attention (CA), and a Strip Pooling Module (SPM). A YUV-guided loss constrained luminance structure, texture gradients, and chrominance to preserve complementary visible and thermal information.
2. We formulated shank-length measurement as localization of the top keypoint (TKP) and bottom keypoint (BKP). An improved YOLOv8s-Pose model used the fused images to predict these endpoints, making the measurement geometry explicit.
3. We added a length-related loss that constrains the distance between predicted endpoints. The resulting pixel distance was converted to physical length and evaluated against manual measurements using correlation, bias, and absolute-error metrics.

## 2. Materials and Methods

### 2.1. Experimental Environment

**Figure 1:**
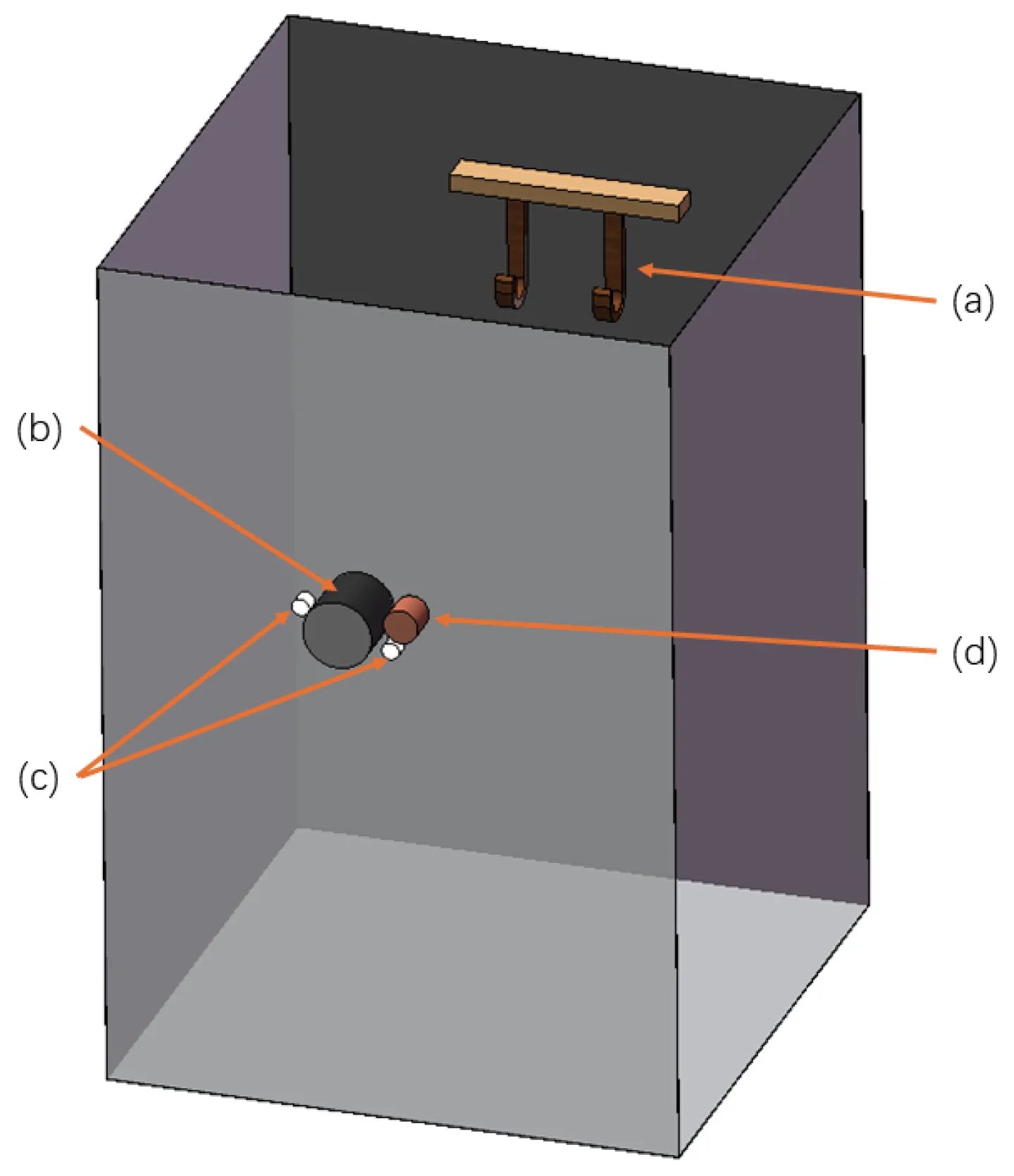
Experimental setup. Labels indicate (a) the restraint hook, (b) the visible-light camera, (c) the custom illumination lamps, and (d) the infrared camera.

The image acquisition platform was constructed with reference to the measurement setup reported by Zheng et al. [1], as shown in Figure 1. An aluminum-profile frame formed the main structure of the platform, and each chicken was suspended and secured using a rigid hook to maintain stability during image acquisition. To obtain complementary texture and thermal information from the shank region, a visible-light camera and an infrared camera were integrated into the same acquisition system. This dual-modal arrangement also helped reduce the effects of feather occlusion and environmental interference on the measurements. Both cameras were positioned facing the plantar side of the suspended chicken’s feet, and their lenses were approximately 380 mm from the rigid hook. A black light-absorbing cloth was placed behind the target area to reduce background interference.

Visible-light images were acquired using a Hikvision MV-CE060-10UC camera (Hangzhou Hikvision Digital Technology Co., Ltd., Hangzhou, China) with a 6 mm focal-length lens and a resolution of 3072×2048 pixels. Infrared images were captured using an IRay AT-61 camera (IRay Technology Co., Ltd., Yantai, China) equipped with a vanadium oxide uncooled infrared focal plane detector. Illumination was provided by two homemade white LED light sources, each arranged in a 3×8 array. One light source was installed to the left of the cameras and the other below the cameras. The angle between the two light sources was $45^{\circ }$. Each light source had a maximum luminous intensity of 738.2 cd and a color temperature of 6000 K, providing relatively uniform and stable illumination during image acquisition. All experimental procedures were conducted in accordance with the guidelines approved by the Experimental Animal Administration and Ethics Committee of South China Agricultural University (SYXK-2024-0136).

### 2.2. Framework Design

**Figure 2:**
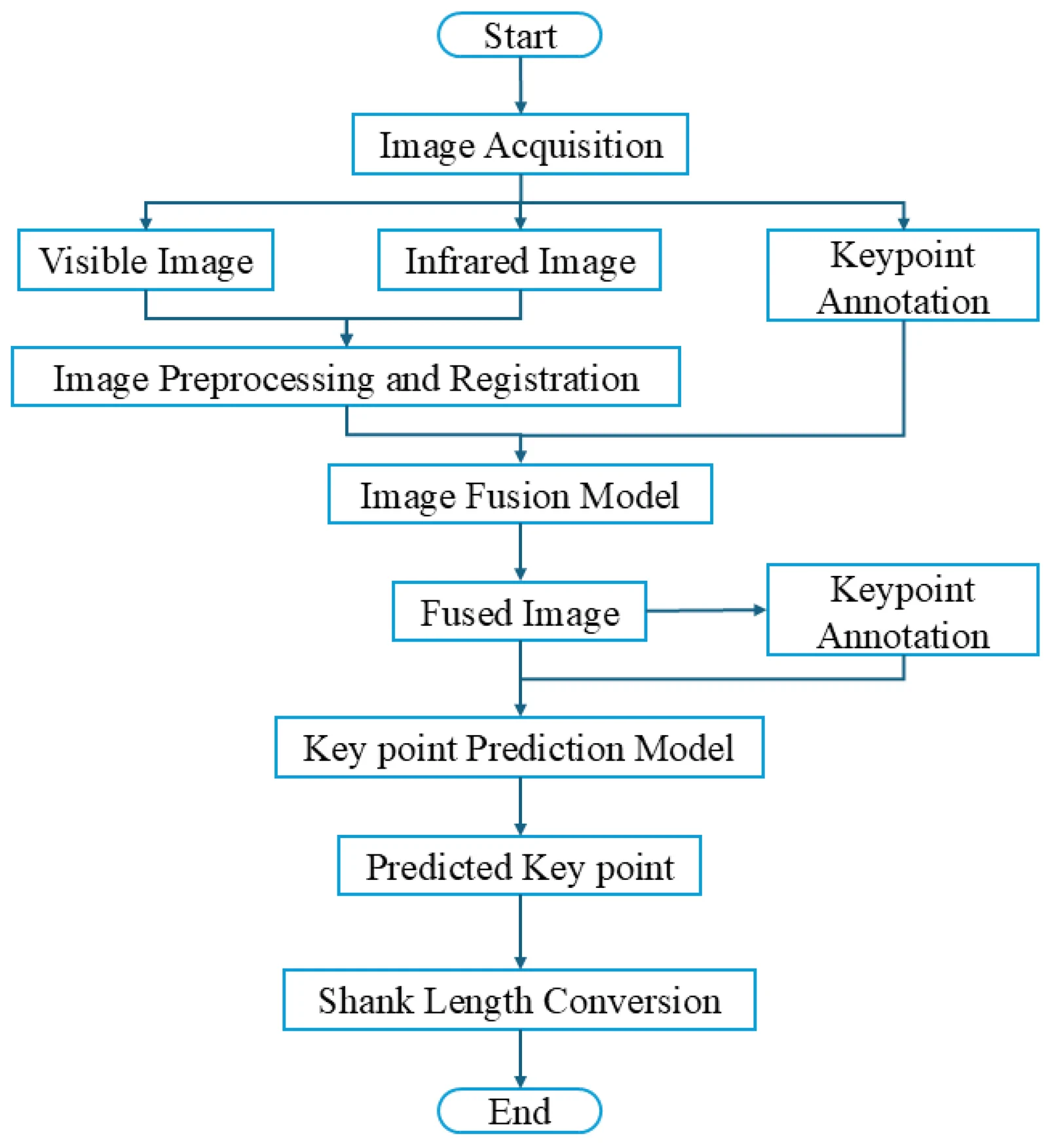
Workflow for image acquisition, visible–infrared registration and fusion, endpoint prediction, and conversion of pixel distance to physical shank length.

A two-stage measurement framework was developed in this study, as shown in Figure 2. First, visible and infrared cameras were used to simultaneously capture dual-modal images of the chicken shank region. The original images were then preprocessed to obtain spatially aligned visible and infrared images. Next, the preprocessed images were fed into the image fusion model to generate fusion images containing texture information from the visible images and thermal feature information from the infrared images.

On this basis, the fusion images were used as input to the keypoint prediction model to predict the two endpoints required for shank length measurement, namely TKP and BKP. Finally, chicken shank length was calculated based on the pixel distance between TKP and BKP and the conversion relationship between pixel distance and actual physical length.

### 2.3. Dataset

Data were collected at the Fuhe Breeder Chicken Farm of Jiangfeng Company in Zengcheng District, Guangzhou, China. The study included 100 live chickens: 50 Mahuang chickens aged 144 or 163 days and 50 yellow-feathered chickens aged 183 days. Ten paired visible and infrared acquisitions were obtained from each bird under the same setup and lighting conditions. The dataset therefore comprised 100 chicken-level groups and 1000 paired acquisitions, corresponding to 1000 visible and 1000 infrared images.

In addition, manual reference shank lengths were obtained using a LINKS electronic digital caliper (Genertec Harbin Measuring & Cutting Tool Co., Ltd., Harbin, China) with a measurement range of 0–150 mm, a display resolution of 0.01 mm, and an accuracy of ±0.03mm. The two anatomical landmarks were defined as the interdigital cleft between the third and fourth toes of the left foot and the depression at the junction between the proximal shank and the feather-covered region. The straight-line distance between these two landmarks was measured. Two operators performed the manual measurements, with each operator measuring each chicken three times, resulting in six readings per chicken. The arithmetic mean of the six readings was used as the chicken-level manual reference shank length. Across the 100 chickens, the manual reference shank lengths ranged from 74.0 to 106.0 mm, with a mean of 87.15 mm. The ten paired visible–infrared acquisitions from the same chicken shared this reference value and were regarded as repeated acquisitions from the same individual rather than independent biological samples.

All images from an individual chicken were assigned to the same data subset to prevent individual-level leakage. Chickens were randomly allocated to training, validation, and test sets at an 8:1:1 ratio. The resulting subsets contained 800 image pairs from 80 chickens, 100 pairs from 10 chickens, and 100 pairs from 10 chickens, respectively. The same individual-level split was used for the fusion and keypoint-prediction experiments.

### 2.4. Image Fusion Model

In chicken shank length measurement, visible images have high resolution and can preserve texture and edge information on feathers, chicken claws, and the shank surface. However, the shank root is easily affected by lighting, feather occlusion, and background interference, resulting in unclear contour boundaries. Infrared images can reflect differences in thermal radiation between the target and the background. They can highlight the thermal distribution of the chicken body and shank regions and provide clear target cues in regions with weak texture. However, infrared images have limited resolution and cannot represent fine texture details effectively. Therefore, by combining the advantages of the two image modalities, a learning-based U-Net-CA-SPM image fusion model was developed for vision-based chicken shank measurement without direct contact with the shank. The model uses an end-to-end learning approach and no longer relies on predefined manual fusion rules. Instead, network training is used to jointly model the texture and structural information from visible images and the thermally salient information from infrared images. In this way, the model learns the complementary relationship between the two types of information and generates fusion images with clear edges, stable target regions, and thermal information representation.

#### 2.4.1. Multi-Source Image Preprocessing and Registration

Before image fusion, the original visible images were first cropped to a region of interest to standardize the model input and highlight useful information in the chicken shank region. Because the visible and infrared cameras differed in resolution, field of view, and imaging position, direct pixel-level fusion would cause misalignment of the chicken shank regions. Therefore, the cropped visible images were used as reference images. Scale transformation, slight rotation, and translation correction were applied to the infrared images so that the chicken body and shank regions in the infrared images could be mapped as closely as possible to the coordinate system of the visible images. After registration and fusion, the resulting fused images had a size of 2176×1632 pixels and served as the coordinate basis for subsequent keypoint prediction, coordinate back-transformation, and physical-length conversion.

**Figure 3:**
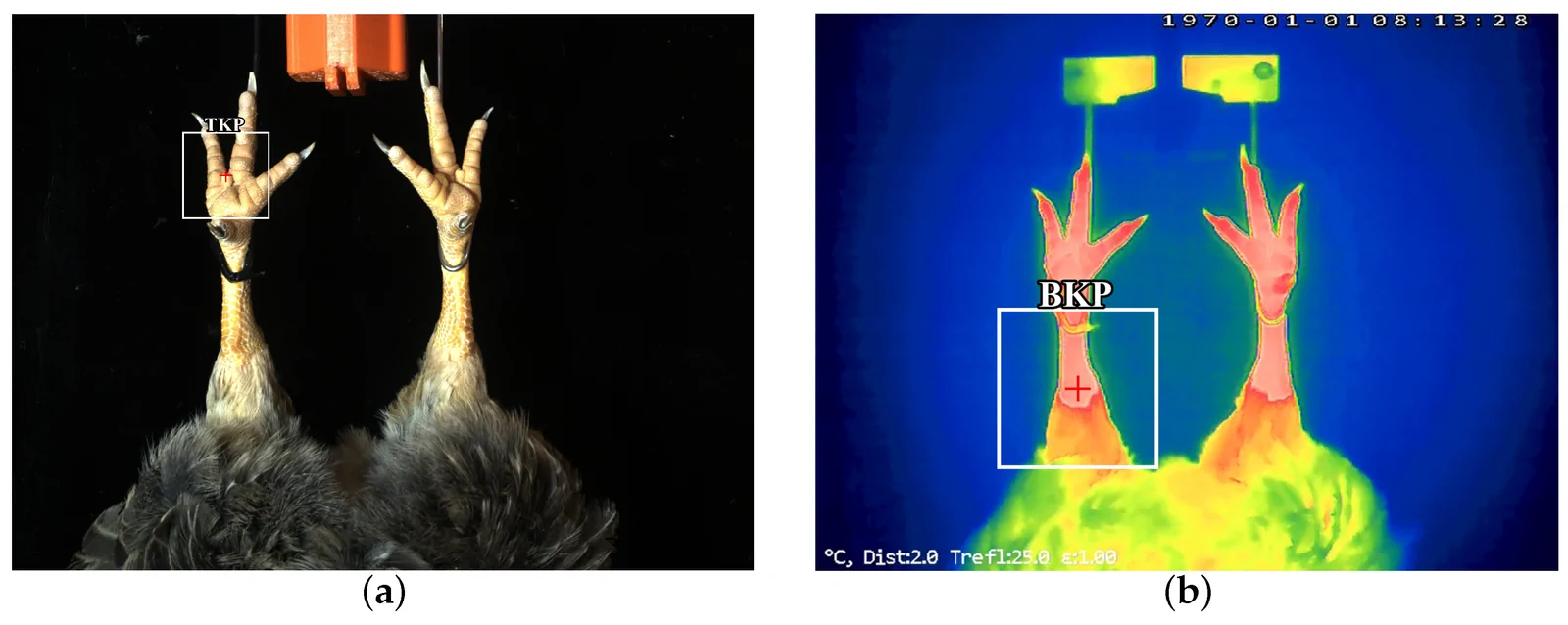
Manual endpoint annotation on the paired source images. (**a**) Top keypoint (TKP) annotated on the visible image; (**b**) bottom keypoint (BKP) annotated on the corresponding infrared image. In subfigure (**b**), the pseudo-color scale represents relative thermal differences, with blue corresponding to cooler regions and red and yellow corresponding to warmer regions.

During annotation of the original dual-modal images, the open-source annotation tool LabelMe (version 5.10.1) was used to determine the keypoint positions according to the advantages of each image modality in representing the shank length endpoints. The gap between the third and fourth toes of the chicken’s left foot showed clear texture and shape features in the visible image. Therefore, this position was annotated in the visible image as the top keypoint (TKP) for shank length measurement. The junction between the visible proximal shank and the feather-covered region of the chicken’s left leg showed a clear thermal boundary in the infrared image. Therefore, this position was annotated in the infrared image as the bottom keypoint (BKP) for shank length measurement. The annotation results were saved in JSON format and matched one to one with the corresponding visible and infrared images. An example of the annotations is shown in Figure 3.

Let P(x,y) denote a pixel in the original infrared image, and let $P^{\prime }\left(x^{\prime },y^{\prime }\right)$ denote the corresponding point in the visible image coordinate system after registration. The affine transformation was performed using Equations (1) and (2).

(1) $$\left[\begin{array}{c} x^{\prime }\\ y^{\prime }\\ 1 \end{array}\right]=M\left[\begin{array}{c} x\\ y\\ 1 \end{array}\right]$$

(2) M=TRS

where P(x,y) represents a pixel in the original infrared image, and $P^{\prime }\left(x^{\prime },y^{\prime }\right)$ represents the corresponding point in the visible image coordinate system after registration. *S*, *R*, and *T* represent the scale transformation matrix, rotation matrix, and translation matrix, respectively. The scale transformation matrix *S* consists of the horizontal scaling factor $s_{x}$ and vertical scaling factor $s_{y}$. The rotation matrix *R* is determined by the rotation angle θ, while the translation matrix *T* consists of the horizontal translation $t_{x}$ and vertical translation $t_{y}$.

A single set of affine parameters was used to map the infrared images to the visible-image coordinate system. The parameters are reported in Table 1.

#### 2.4.2. Pseudo-Color Enhancement of Infrared Images

The original infrared images are single-channel grayscale thermal images. In grayscale form, their ability to represent thermal distribution features is relatively limited, making it difficult to highlight differences in thermal distribution within the chicken shank region during image fusion. Therefore, the registered infrared grayscale images were normalized using Equation (3). Their pixel values were mapped to the range of 0 to 255 to increase the grayscale contrast between regions with different temperatures.

(3) $$I_{\mathrm{norm}}\left(x,y\right)=255\cdot \frac{I_{\mathrm{ir}}\left(x,y\right)-I_{\min}}{I_{\max}-I_{\min}}$$

where $I_{\mathrm{norm}}\left(x,y\right)$ is the normalized pixel value of the infrared image, and $I_{\mathrm{ir}}\left(x,y\right)$ is the pixel value of the registered infrared grayscale image. $I_{\min}$ and $I_{\max}$ are the minimum and maximum grayscale values in the infrared image, respectively. *x* and *y* represent the horizontal and vertical coordinates of an image pixel, respectively. The constant 255 indicates that the normalized result is linearly mapped to the grayscale range [0,255] of an 8-bit grayscale image.

Next, the Jet pseudo-color mapping in Equation (4) was used to convert the single-channel infrared images into three-channel color images. This mapping represents low-temperature regions in blue and high-temperature regions in red or yellow, making the thermally salient information in the chicken body and shank regions more clearly visible. This representation helps the network learn the thermal differences between the target region and the background. It also allows the infrared and visible images to be modeled in a consistent three-channel space. Therefore, the pseudo-color infrared images were used as the thermal information input to the fusion network to improve the model’s ability to represent thermal boundaries and thermally salient features in the chicken shank region.

(4) $$I_{\mathrm{pc}}\left(x,y\right)=C_{\mathrm{Jet}}\left(I_{\mathrm{norm}}\left(x,y\right)\right)$$

where $I_{\mathrm{pc}}\left(x,y\right)$ is the three-channel color representation of the pseudo-color infrared image at (x,y), and $C_{\mathrm{Jet}}\left(\cdot \right)$ is the Jet pseudo-color mapping function.

#### 2.4.3. Fusion Network Architecture

The U-Net model was proposed by Ronneberger et al. [20] in 2015. Based on a fully convolutional network, U-Net uses an encoder–decoder architecture composed of a contracting path and a symmetric expanding path. It concatenates high-resolution features from the encoder with upsampled features from the decoder, allowing the model to obtain contextual information while maintaining precise localization. Compared with traditional sliding-window segmentation methods, U-Net reduces repeated computation and is more suitable for pixel-level segmentation tasks. ResNet was proposed by He et al. [21] in 2016. It uses identity shortcut connections to construct residual learning units. As the network becomes deeper, this design helps alleviate vanishing gradients and network degradation, thereby improving feature extraction and training stability. Hou et al. [22] proposed the Coordinate Attention module. This module separately aggregates features along the two spatial directions and generates coordinate-aware attention weights. It preserves precise positional information while capturing long-range dependencies in lightweight mobile networks, thereby improving performance in image classification, object detection, and semantic segmentation. Hou et al. [23] proposed the Strip Pooling module. This module uses strip-shaped pooling windows along one spatial direction to capture long-range contextual information while preserving local details along the other direction. It improves the network’s ability to model strip-shaped structures and regions with discrete distributions, thereby improving scene parsing performance in complex scenes.

**Figure 4:**
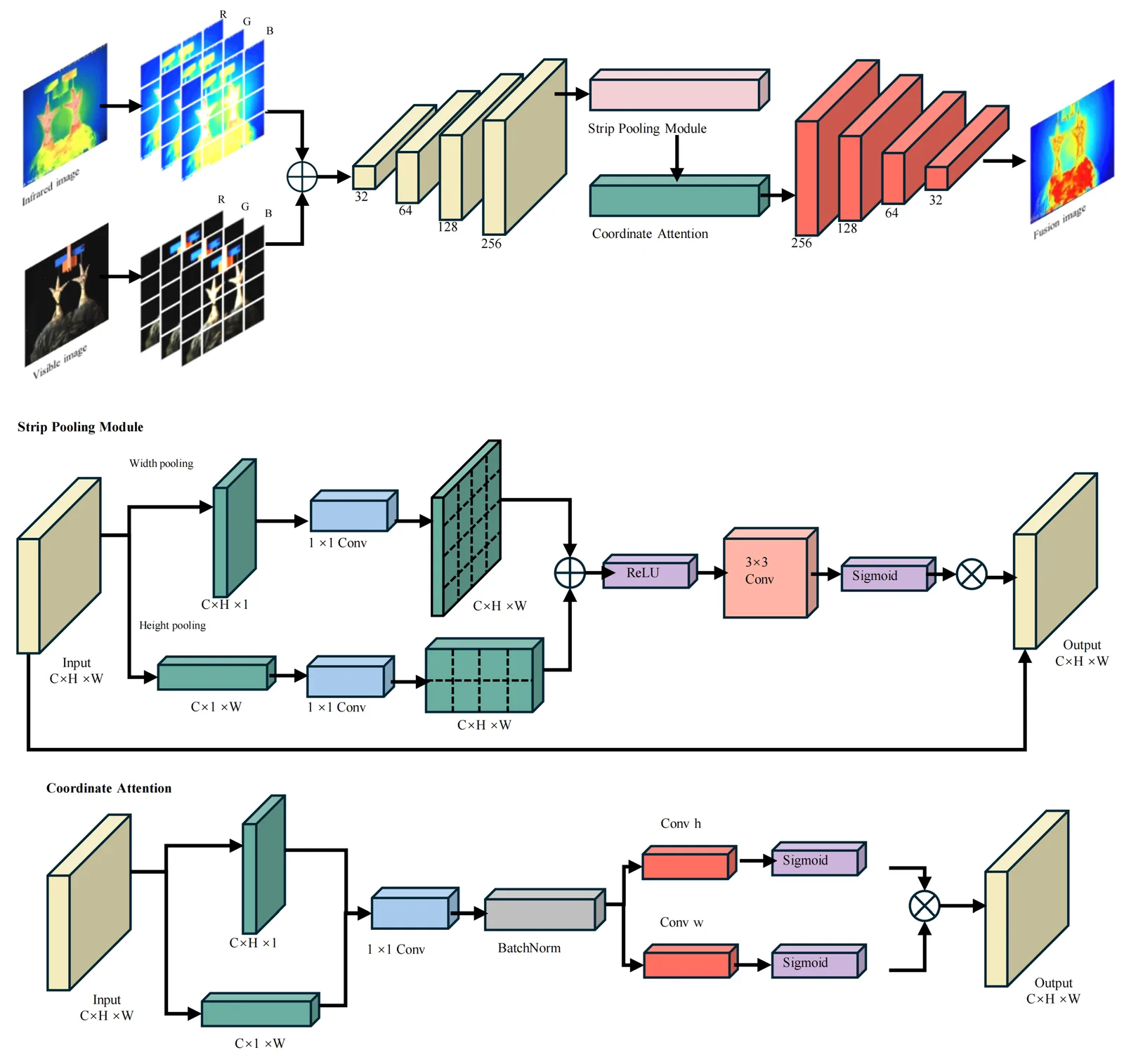
Architecture of U-Net-CA-SPM for visible–infrared image fusion. CA denotes Coordinate Attention, and SPM denotes the Strip Pooling Module. Different colors are used to visually distinguish the network components and feature-processing operations and do not represent quantitative values.

The fusion network developed in this study is based on a U-Net-style encoder–decoder architecture, as shown in Figure 4. Residual modules were embedded in the encoder to alleviate the degradation problem during training and improve multiscale feature extraction. To strengthen the spatial information of the features and the responses to key regions, the Strip Pooling module and the Coordinate Attention module were introduced in sequence into the bottleneck layer at the end of the encoder. During decoding, skip connections were used to combine shallow detail features with deep semantic features, thereby improving the joint representation of texture information from visible images and thermal information from infrared images.

#### 2.4.4. YUV-Guided Loss Function

Infrared and visible images focus on different types of information. Infrared images are effective at representing thermal targets and intensity distributions, while visible images preserve more textures, edges, and details. Directly applying an overall constraint to the fusion result in the RGB color space may result in unclear structural and color information in the fusion image. Yu et al. [24] separated the luminance and chrominance components in the ${\mathrm{YC}}_{b}C_{r}$ color space, reducing color distortion and oversaturation in pseudo-color image fusion. Zhuang et al. [25] further focused on color preservation during image fusion and improved the color naturalness of the fusion results by processing intensity and color information separately.

Based on these studies, a YUV-guided loss function was designed to supervise U-Net-CA-SPM and improve the structural clarity and thermal information representation of the fusion images. The fusion constraints were applied in the YUV color space. The *Y* channel was used to constrain luminance and texture, while the *U* and *V* channels were used to constrain the color representation of the pseudo-color infrared images. The specific equations are as follows.

(5) $$L_{Y}^{pix}={∥Y_{f}-\max\left(Y_{vis},I_{ir}\right)∥}_{1}$$

(6) $$L_{Y}^{grad}={∥\nabla Y_{f}-\max\left(\nabla Y_{vis},\nabla I_{ir}\right)∥}_{1}$$

(7) $$L_{UV}^{pix}={∥\left[U_{f},V_{f}\right]-\left[U_{ir}^{c},V_{ir}^{c}\right]∥}_{1}$$

(8) $$L_{UV}^{grad}={∥\nabla U_{f}-\nabla U_{ir}^{c}∥}_{1}+{∥\nabla V_{f}-\nabla V_{ir}^{c}∥}_{1}$$

(9) $$L=\lambda_{1}L_{Y}^{pix}+\lambda_{2}L_{Y}^{grad}+\lambda_{3}L_{UV}^{pix}+\lambda_{4}L_{UV}^{grad}$$

where $L_{Y}^{pix}$ represents the luminance pixel loss, $L_{Y}^{grad}$ represents the luminance gradient loss, $L_{UV}^{pix}$ represents the chrominance pixel loss, and $L_{UV}^{grad}$ represents the chrominance gradient loss. ∇ denotes the Sobel gradient operator. The weighting coefficients of the four loss terms are represented by $\lambda_{1}$, $\lambda_{2}$, $\lambda_{3}$, and $\lambda_{4}$, respectively.

### 2.5. Keypoint Prediction Method for Chicken Shank Length Measurement

**Figure 5:**
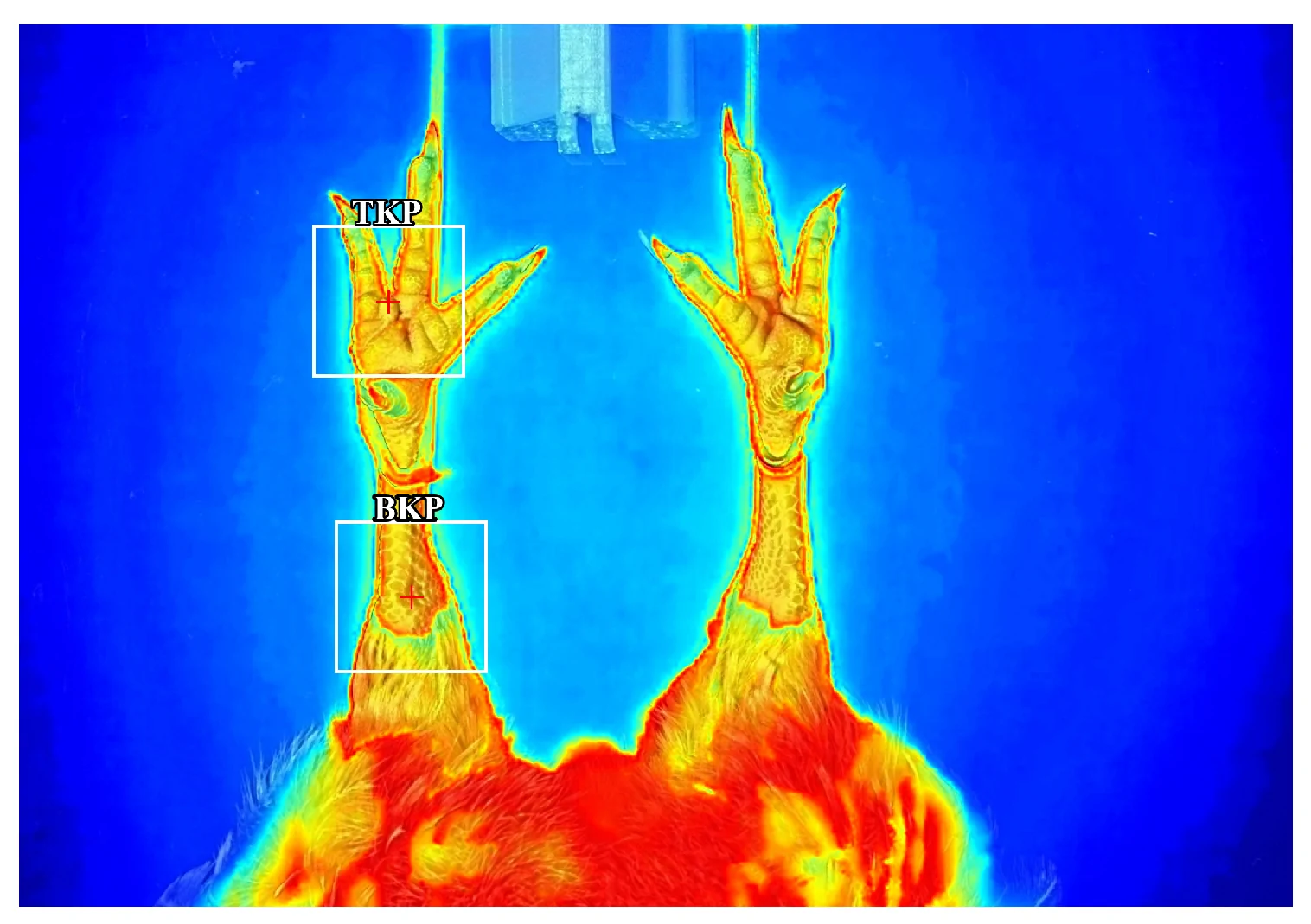
Manual annotation of the top keypoint (TKP) and bottom keypoint (BKP) on a fused image. The red crosses indicate the manually annotated keypoint locations, and the white boxes indicate the corresponding endpoint regions. The pseudo-color appearance of the fused image retains thermal information, with warmer regions predominantly shown in red and yellow and cooler regions predominantly shown in blue.

Before training the keypoint prediction model, the labels of the shank length endpoints were recreated based on the fusion images. The fusion images differ from the single-modality images in their representation of textures, edges, and thermal information. Therefore, to ensure that the labels used to supervise the keypoint prediction model were consistent with the input images, TKP and BKP were manually reannotated on each fusion image using LabelMe, as shown in Figure 5. The annotation results were saved as JSON files. The fusion images and their corresponding JSON labels were then converted into the data format required by YOLOv8s-Pose for training, validation, and testing of the keypoint prediction model.

**Figure 6:**
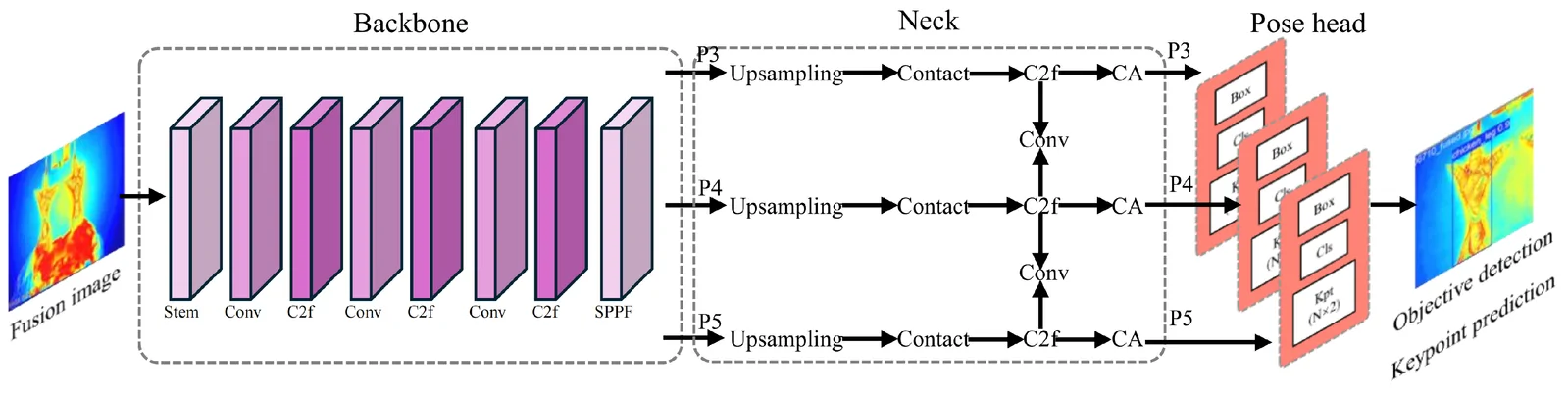
Architecture of YOLOv8s-Pose-CA for localization of the top and bottom shank endpoints. CA denotes Coordinate Attention. Purple blocks indicate the backbone feature-extraction modules, while red blocks indicate the pose head. The colors are used to visually distinguish different architectural stages and do not represent quantitative values.

For the chicken shank length measurement task, this study developed a keypoint prediction model based on an improved YOLOv8s-Pose, as shown in Figure 6. The model takes the fused chicken images as input and uses the improved YOLOv8s-Pose to predict the chicken shank keypoints, producing the coordinates of TKP and BKP. On this basis, a shank length-related constraint was introduced into the original keypoint detection loss of YOLOv8s-Pose. This constraint allows the model to further consider the distance relationship between the two points while optimizing their coordinates.

Zheng et al. [1] developed a measurement procedure consisting of region of interest (ROI) extraction, keypoint extraction, and length calculation. They clearly stated that both shank length and shank circumference were calculated based on keypoint information, with shank length determined by TKP and BKP. This indicates that keypoint localization is an essential basis for the subsequent length calculation. Therefore, the keypoint prediction model developed in this study was not intended for general pose estimation. Instead, it converted chicken shank length measurement into a problem of locating two measurement endpoints. First, the process of calculating length from the keypoints is more direct, and the model output has a clear geometric meaning. Second, stable and accurate keypoint prediction can improve the stability and accuracy of the subsequent shank length calculation, thereby reducing variation in the measurement results.

#### 2.5.1. YOLOv8s-Pose

YOLOv8s-Pose is derived from the YOLOv8 object detection framework and is a lightweight keypoint detection model designed for human pose estimation. In this study, its ability to perform object localization and keypoint detection simultaneously was used to convert the chicken shank length measurement task into the localization of the upper and lower endpoints of the chicken shank. However, the original YOLOv8s-Pose model was developed mainly for human pose estimation. It has certain limitations when applied to chicken shank length measurement because of the slender target structure, feather occlusion, and unclear local boundaries.

To improve the model’s ability to represent key regions of the chicken shank, the Coordinate Attention module was also introduced into the keypoint prediction model. It should be noted that, in the first-stage fusion model, the Coordinate Attention module enhances the representation of useful information during multi-source image feature fusion. In contrast, in the keypoint prediction model, it strengthens the model’s attention to the slender structure and key regions of the chicken shank. Thus, the module has different functions and purposes in the two models. Based on this, the Coordinate Attention module was introduced during the feature fusion stage of YOLOv8s-Pose to improve the model’s ability to capture features around the keypoints, reduce interference caused by feathers, and improve keypoint localization accuracy.

#### 2.5.2. Supervision with a Shank Length-Related Constraint

Ji et al. [26] introduced the distance and direction differences between adjacent keypoints into the loss function. These differences were used to constrain the relative positions between the predicted and ground-truth keypoints, thereby improving the structural validity of the two-dimensional human pose estimation results. Liu et al. [27] proposed Bone Loss based on the SimCC coordinate representation. This loss supervises the length relationships between human bone segments by comparing the predicted and ground-truth lengths of the bone segments formed by connected keypoints. They also proposed Sum Loss to constrain the number of visible keypoints.

In this study, using only the original keypoint detection loss of YOLOv8s-Pose would provide no direct constraint on the length consistency between the two points. As a result, the coordinate error of an individual keypoint could propagate to the calculated shank length. Therefore, a shank length-related constraint constructed from the distance between TKP and BKP was further introduced during YOLOv8s-Pose training, as shown in Equation (10). This constraint allows the model to consider the consistency of the represented shank length while optimizing the keypoint coordinates, thereby improving the stability and accuracy of the prediction results.

(10) $$\left\{\begin{array}{cc} L & =L_{YOLOpose}+0.20L_{len,MAE}+0.10L_{len,RMSE}\\ L_{len,MAE} & =\frac{1}{N}\sum_{i=1}^{N}\left|{\hat{l}}_{i}-l_{i}\right|\\ L_{len,RMSE} & =\sqrt{\frac{1}{N}\sum_{i=1}^{N}{\left({\hat{l}}_{i}-l_{i}\right)}^{2}+\varepsilon } \end{array}\right.$$

where $L_{YOLOpose}$ is the original keypoint detection loss of YOLOv8s-Pose. $L_{len,MAE}$ is the mean absolute error term between the predicted and annotated shank segment lengths, while $L_{len,RMSE}$ is the corresponding root mean square error term. *N* represents the number of samples included in the loss calculation, and ε is a very small constant introduced to prevent numerical errors caused by zero values. In addition, ${\hat{l}}_{i}$ and $l_{i}$ represent the pixel lengths of the shank segments formed by the predicted and annotated keypoints in the *i*-th sample, respectively. They are calculated using Equation (11).

(11) $$\left\{\begin{array}{cc} {\hat{l}}_{i} & =\sqrt{{\left({\hat{X}}_{t,i}-{\hat{X}}_{b,i}\right)}^{2}+{\left({\hat{Y}}_{t,i}-{\hat{Y}}_{b,i}\right)}^{2}}\\ l_{i} & =\sqrt{{\left(X_{t,i}-X_{b,i}\right)}^{2}+{\left(Y_{t,i}-Y_{b,i}\right)}^{2}} \end{array}\right.$$

where $\left({\hat{X}}_{t,i},{\hat{Y}}_{t,i}\right)$ and $\left({\hat{X}}_{b,i},{\hat{Y}}_{b,i}\right)$ represent the predicted coordinates of TKP and BKP, respectively. $\left(X_{t,i},Y_{t,i}\right)$ and $\left(X_{b,i},Y_{b,i}\right)$ represent the annotated coordinates of TKP and BKP, respectively.

### 2.6. Model Parameter Settings

The principal training settings are summarized in Table 2. All experiments used the individual-level split described in Section 2.3. Training was performed on one NVIDIA GeForce RTX 4090 D GPU (NVIDIA Corporation, Santa Clara, CA, USA) with 24 GB of memory using PyTorch 2.6.0+cu124 and CUDA 12.4. Within each comparison, the data split and evaluation procedure were held constant; architecture, input modality, loss function, or keypoint framework varied according to the experimental question.

### 2.7. Evaluation Methods

During inference, the trained YOLOv8s-Pose-CA model was used to predict the TKP and BKP coordinates for each test image. Each fused image used for keypoint prediction had a size of 2176×1632 pixels. A centered 1536×1536 coarse crop was first extracted for shank-region detection. The detected bounding box was then mapped back to the 2176×1632 fused-image coordinate system, and a square fine crop centered on the detected shank region was generated. The side length of the fine crop was set to 1.5 times the larger of the detected bounding-box width and height. When the square fine crop exceeded an image boundary, the crop window was shifted so that the entire square remained within the image while retaining its side length. The fine crop was directly resized to 1024×1024 by interpolation without letterbox padding. Therefore, the TKP and BKP coordinates predicted in the 1024×1024 model-input coordinate system were mapped back to the fused-image coordinate system using the scale factor and fine-crop offsets defined in Equation (12).

(12) $$\left\{\begin{array}{cc} s & =\frac{c}{1024},\\ X & =x\;s+x_{0},\\ Y & =y\;s+y_{0} \end{array}\right.$$

where *c* is the side length of the fine crop, (x,y) denotes a predicted coordinate in the resized model input, and $\left(x_{0},y_{0}\right)$ denotes the upper-left corner of the fine crop in the 2176×1632 fused image. The Euclidean distance between the back-transformed TKP and BKP coordinates was then converted to physical shank length using Equation (13).

(13) $$L_{mm}=\sqrt{{\left(X_{t}-X_{b}\right)}^{2}+{\left(Y_{t}-Y_{b}\right)}^{2}}\cdot CF$$

where $L_{mm}$ represents the converted predicted shank length. $\left(X_{t},Y_{t}\right)$ and $\left(X_{b},Y_{b}\right)$ represent the TKP and BKP coordinates after back-transformation to the 2176×1632 fused-image coordinate system, respectively. CF represents the conversion factor between pixel length and physical length.

**Figure 7:**
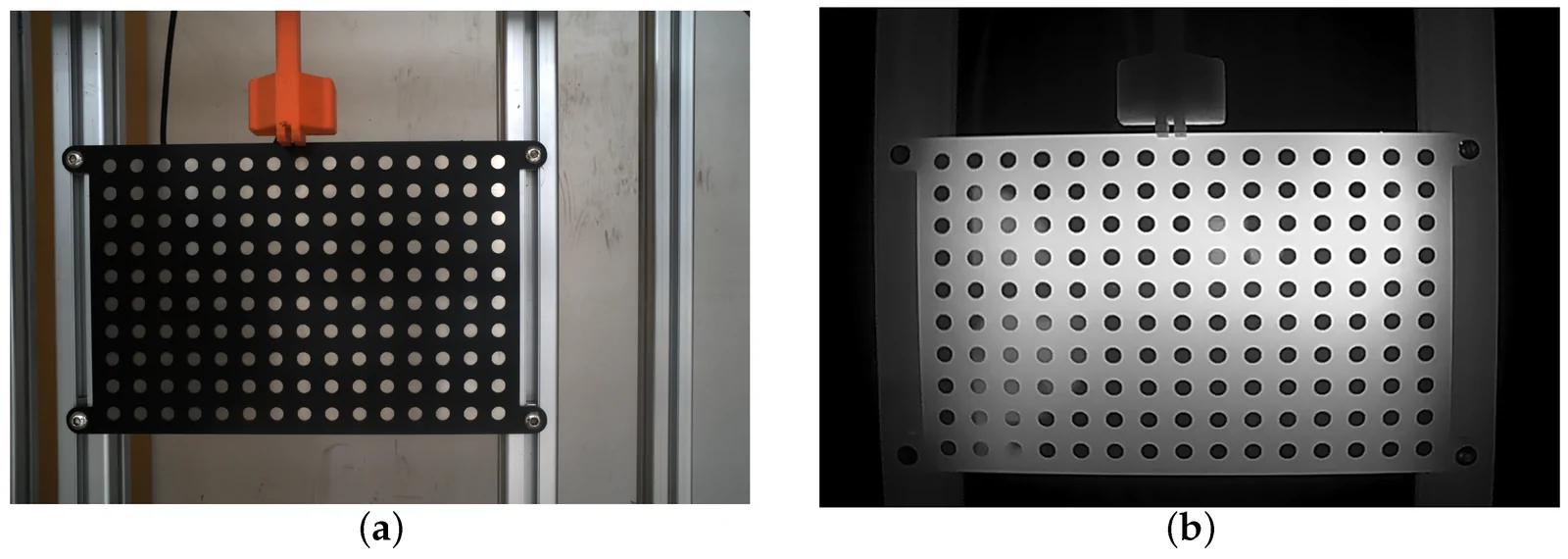
Paired visible and infrared images of the calibration board used to estimate the pixel-to-length conversion factor and to evaluate visible–infrared registration accuracy. (**a**) Visible image; (**b**) infrared image. Adjacent hole centers are separated by 20 mm.

The conversion factor CF used in this study was calculated using the calibration board shown in Figure 7 and Equation (14). The known physical distance between the centers of two adjacent circular holes on the calibration board was 20 mm. LabelMe was used to annotate the centers of the corresponding circular holes in the visible image of the calibration board, thereby obtaining their pixel coordinates in the image coordinate system.

(14) $$CF=\frac{D_{real}}{d_{px}}$$

where CF is the conversion factor, $D_{real}$ is the physical distance between the two points, and $d_{px}$ is the Euclidean distance between the two pixel coordinates. To reduce random errors caused by a single calibration, two pairs of circular hole centers with different physical distances were selected for calibration. The first pair consisted of two adjacent hole centers with a physical distance of 20mm. The second pair consisted of two hole centers located farther apart, with a physical distance of 80mm. Finally, the mean of the two calibration results was used as the conversion factor for the shank length measurement stage. Thus, CF was set to 0.190367 mm/px for measurements in the 2176×1632 fused-image coordinate system.

To quantitatively evaluate the accuracy of the fixed affine transformation, one paired visible–infrared acquisition of the calibration board shown in Figure 7 was used. A total of 150 corresponding circular-hole centers (10 × 15) were included. The infrared hole centers were mapped to the visible-image coordinate system using the same fixed affine transformation described above, and the Euclidean distance between each transformed infrared hole center and its corresponding visible hole center was calculated as the landmark registration error. The mean registration error, RMSE, 95th-percentile error, and maximum error were calculated across all 150 landmark pairs. The pixel errors were also converted to physical distances using the conversion factor CF=0.190367mm/px defined above.

Endpoint localization accuracy was evaluated on the 100 fused test images using the back-transformed coordinates. For TKP and BKP separately, Euclidean pixel errors between the predicted and manually annotated coordinates were calculated. The mean, median, and 95th-percentile pixel errors were reported, together with the mean normalized error, which was defined as the endpoint localization error divided by the annotated TKP–BKP pixel distance in the corresponding image.

Because each chicken contributed ten repeated paired acquisitions, observations from the same chicken were not treated as independent biological samples. For the primary measurement-performance analysis, the ten predicted shank lengths from each chicken were summarized by their median to obtain one representative prediction for that chicken. This value was then compared with the corresponding manual reference shank length, and the individual chicken was used as the independent analysis unit.

For comparisons of the fused-image input with each single-modality input, paired chicken-level absolute errors were compared using two-sided Wilcoxon signed-rank tests with Holm correction for the two pairwise comparisons, and statistical significance was set at p<0.05.

Pearson’s correlation coefficient $R_{X,Y}$ was used to quantify the linear association between model-derived and manual reference measurements. Following Schober et al. [28], absolute coefficients below 0.10, from 0.10 to below 0.40, from 0.40 to below 0.70, from 0.70 to below 0.90, and at least 0.90 were interpreted as negligible, weak, moderate, strong, and very strong correlations, respectively. Because correlation does not measure agreement, mean absolute error (MAE), root mean square error (RMSE), and mean signed error (Bias) were also calculated. Within-chicken repeatability was evaluated separately from agreement with the manual reference measurement. For each test chicken, SD, CV, and floating error $f_{e}$ were calculated from the ten repeated model-derived shank lengths.

(15) $$R_{X,Y}=\frac{\mathrm{cov}(X,Y)}{\sigma_{X}\sigma_{Y}}$$

where cov(X,Y) represents the covariance between the two variables, and $\sigma_{X}$ and $\sigma_{Y}$ represent the standard deviations of *X* and *Y*, respectively.

(16) $$MAE=\frac{1}{N}\sum_{i=1}^{N}\left|L_{i}-L_{i}^{0}\right|$$

(17) $$RMSE=\sqrt{\frac{1}{N}\sum_{i=1}^{N}{\left(L_{i}-L_{i}^{0}\right)}^{2}}$$

(18) $$Bias=\frac{1}{N}\sum_{i=1}^{N}\left(L_{i}-L_{i}^{0}\right)$$

where $L_{i}$ is the representative model-derived shank length for chicken *i*, obtained as the median of its ten repeated predictions; $L_{i}^{0}$ is the corresponding manual reference shank length; and *N* is the number of individual chickens included in the analysis. For the test-set analysis, N=10.

(19) $$SD=\sqrt{\frac{1}{K}\sum_{j=1}^{K}{\left(L_{j}-\bar{L}\right)}^{2}}$$

(20) $$CV=\frac{SD}{\bar{L}}\times 100\%$$

(21) $$f_{e}=\frac{L_{\max}-L_{\min}}{L_{\min}}\times 100\%$$

For Equations (19)–(21), $L_{j}$ denotes the model-derived shank length from the *j*th repeated acquisition of a given chicken, $\bar{L}$ denotes the mean of the ten repeated predictions, and K=10 is the number of repeated acquisitions for each chicken. $L_{\max}$ and $L_{\min}$ denote the maximum and minimum predicted shank lengths among those ten acquisitions, respectively. The SD, CV, and $f_{e}$ values were calculated separately for each test chicken and then summarized as the mean and range across the ten test chickens. The 95th-percentile absolute error (P95) and maximum absolute error were defined as

(22) $$P95=Q_{0.95}\left({\left\{\left|L_{i}-L_{i}^{0}\right|\right\}}_{i=1}^{N}\right)$$

(23) $$E_{\max}=\max_{1\leq i\leq N}\left|L_{i}-L_{i}^{0}\right|$$

where $Q_{0.95}$ denotes the empirical 95th percentile.

## 3. Results

### 3.1. Registration Accuracy and Image Fusion Results

The fixed affine transformation yielded a mean landmark registration error of 0.835 px (0.159 mm) and an RMSE of 0.961 px (0.183 mm). The 95th-percentile and maximum errors were 1.865 px (0.355 mm) and 2.936 px (0.559 mm), respectively. Thus, the mean landmark misalignment for the calibration–board image pair used in this evaluation was below one pixel.

**Figure 8:**
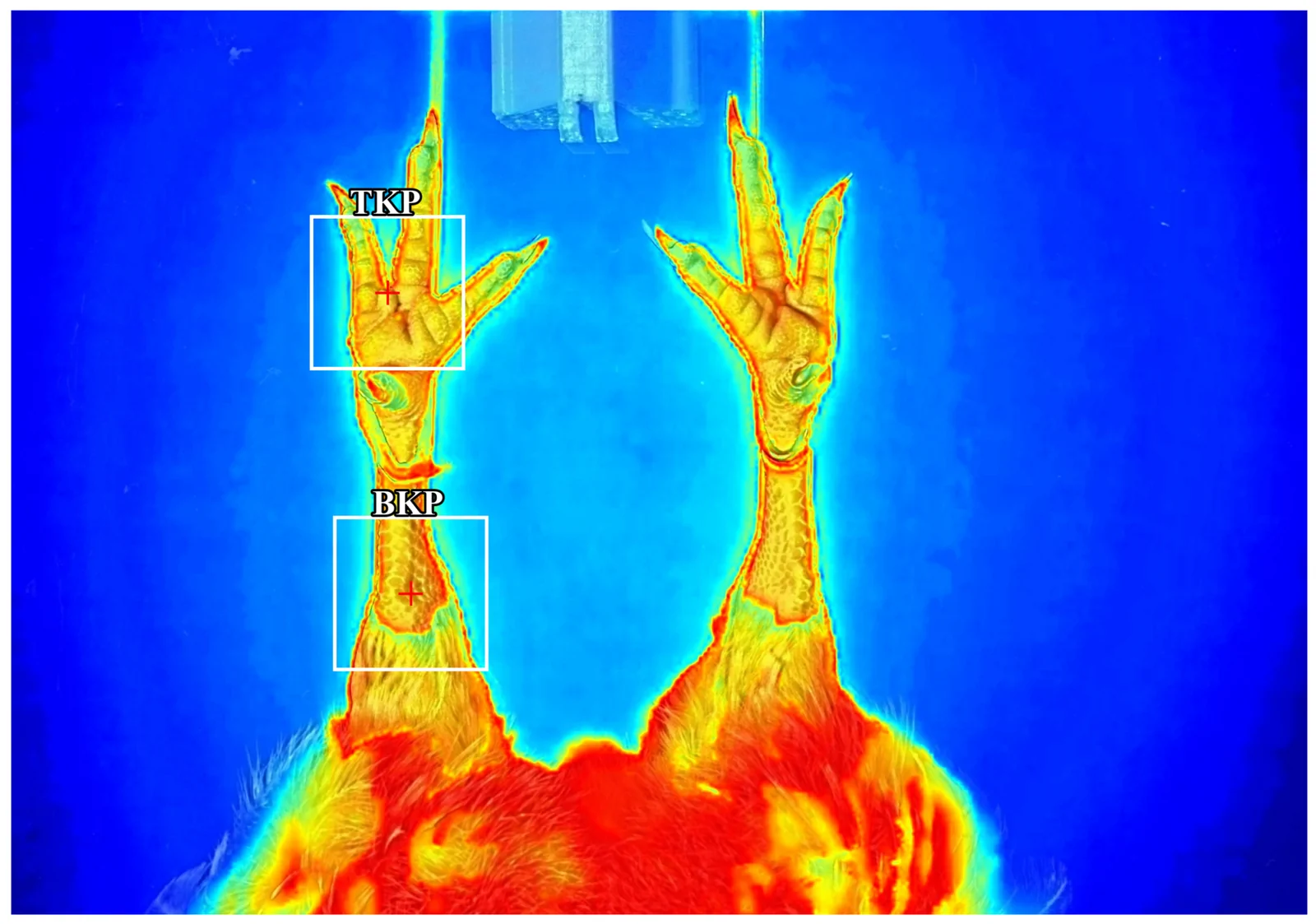
Representative output of the visible–infrared image-fusion model, with the two endpoint regions indicated.

The fusion results obtained using the image fusion model described in Section 2.4 are shown in Figure 8. The visible images clearly present the texture and edge features of the chicken claw region and shank surface. However, the boundary of the shank root is difficult to distinguish because of feather occlusion. As shown in Figure 8, after pseudo-color mapping, the low-temperature background in the infrared images is mainly blue, while exposed regions with higher temperatures, such as the chicken shank, are mainly red. The edges of the feather-covered regions and the temperature transition regions appear green to yellow. These color differences effectively highlight the contours of the chicken legs and reduce interference caused by feathers. However, compared with the visible images, the infrared images lack many texture details.

Therefore, the fusion model can use the texture and edge information from visible images and the thermal boundary information from infrared images in a complementary manner. This improves the distinguishability of the chicken shank root and the regions near the measurement endpoints, providing a more stable image basis for the subsequent localization of TKP and BKP.

### 3.2. Keypoint Prediction and Shank Length Measurement Results

**Figure 9:**
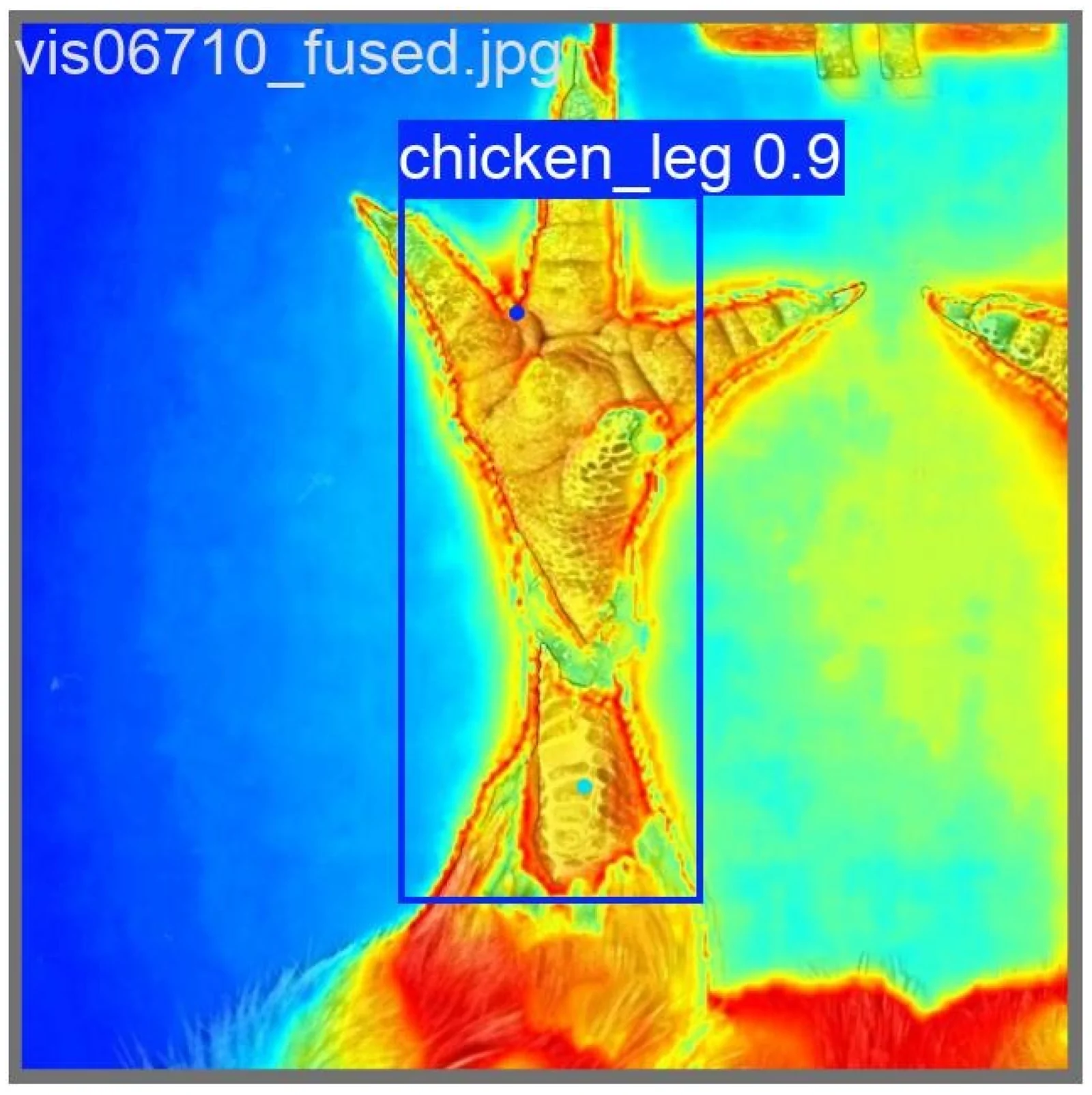
Representative keypoint-prediction output. The blue bounding box identifies the detected shank region. The dark-blue marker indicates the predicted top keypoint (TKP), and the cyan marker indicates the predicted bottom keypoint (BKP).

The keypoint model was evaluated on the test set, which contained 100 paired acquisitions from 10 chickens. Figure 9 provides a qualitative example in which the predicted keypoints were close to their manual annotations. The endpoint localization errors of TKP and BKP on the 100 fused test images are summarized in Table 3.

TKP showed a mean localization error of 8.373 px and a mean normalized error of 1.354%, whereas BKP showed a mean localization error of 10.566 px and a mean normalized error of 1.691%. The 95th-percentile localization error was 13.958 px for TKP and 27.384 px for BKP. The larger localization errors observed for BKP suggest that the proximal endpoint near the feather-covered shank boundary was more difficult to localize consistently than the interdigital endpoint.

**Figure 10:**
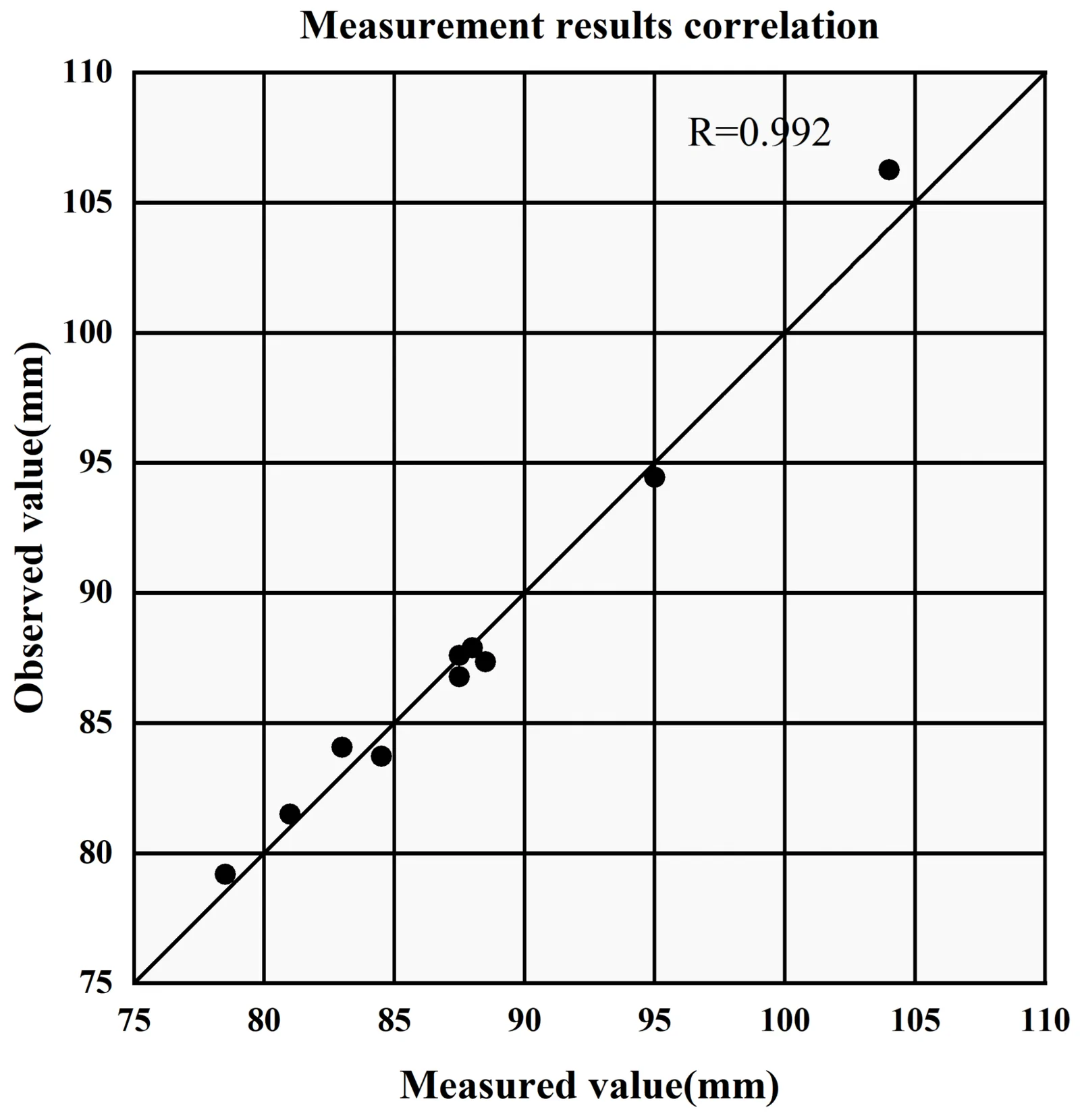
Chicken-level comparison between model-derived and manual reference shank lengths. Each point represents one chicken, and the model-derived value is the median of ten repeated predictions. The solid diagonal line is the identity line, and R denotes the Pearson correlation coefficient.

After evaluating endpoint localization at the acquisition level, shank-length agreement was assessed at the chicken level to account for the repeated acquisitions from each individual. For each chicken, the ten predicted shank lengths were summarized by their median and compared with the corresponding manual reference value. The Pearson correlation coefficient between the model-derived and manual reference shank lengths was R=0.992. Figure 10 shows the corresponding chicken-level comparison.

**Figure 11:**
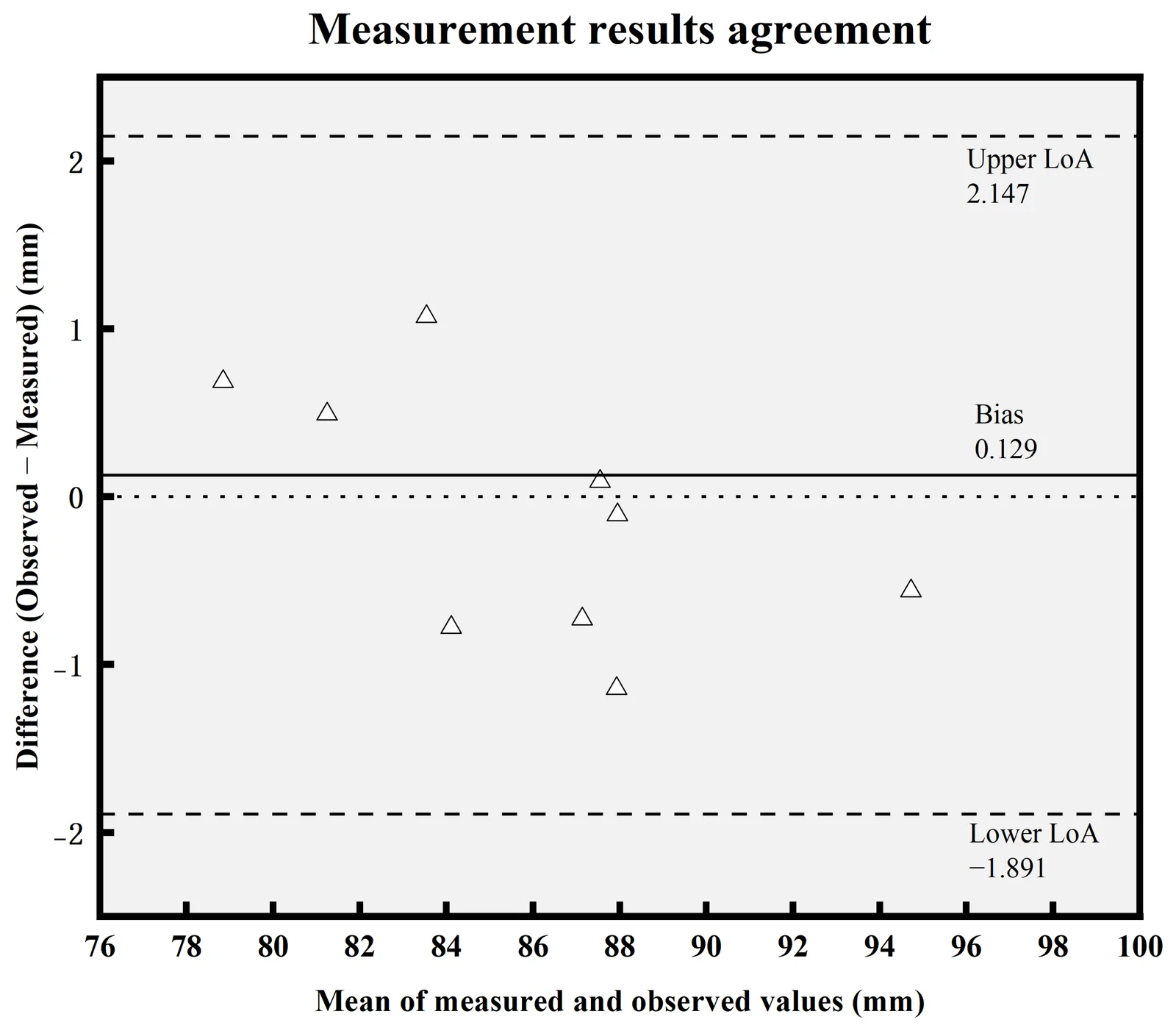
Chicken-level Bland–Altman plot comparing model-derived and manual reference shank lengths. Each point represents one chicken, and the model-derived value is the median of ten repeated predictions. The central line shows the reported mean difference (0.129 mm), and the outer lines show the 95% limits of agreement (2.147 and −1.891 mm).

As shown in Figure 11 and Table 4, the chicken-level systematic bias was 0.129 mm, indicating that the median model-derived shank lengths showed only a slight tendency to be overestimated compared with the manual reference values. When considered together with the MAE of 0.790 mm and RMSE of 0.984 mm, these results indicate close agreement between the model-derived and manual reference shank lengths at the chicken level.

The 95th-percentile absolute error was 1.751 mm, and the maximum absolute error was 2.249 mm.

In addition to the chicken-level agreement analysis, within-chicken repeatability was evaluated using the ten repeated model-derived shank lengths for each test chicken, and the resulting metrics are summarized in Table 5.

Across the ten test chickens, the mean within-chicken SD was 0.716 mm, the mean within-chicken CV was 0.815%, and the mean within-chicken floating error was 2.528%. Together, these results indicate limited within-chicken variability across repeated acquisitions under the controlled acquisition conditions, supporting the repeatability of the proposed measurement method.

## 4. Discussion

### 4.1. Comparison Experiments Using Different Data Sources

**Figure 12:**
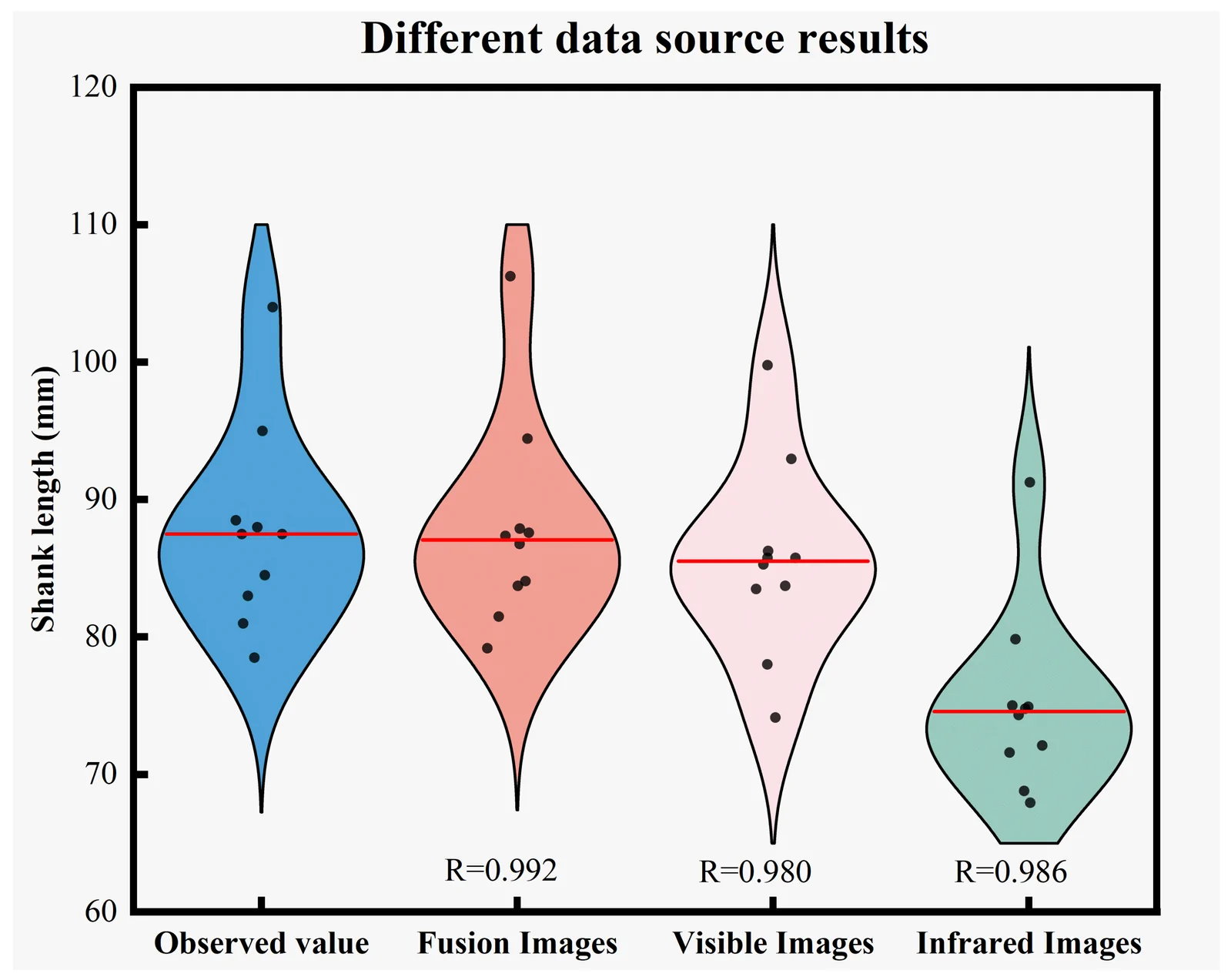
Distributions of manual and model-derived shank lengths obtained with visible-only, infrared-only, and fused-image inputs. R denotes the Pearson correlation coefficient between model-derived and manual measurements. The red horizontal lines indicate the median values of the corresponding distributions.

**Figure 13:**
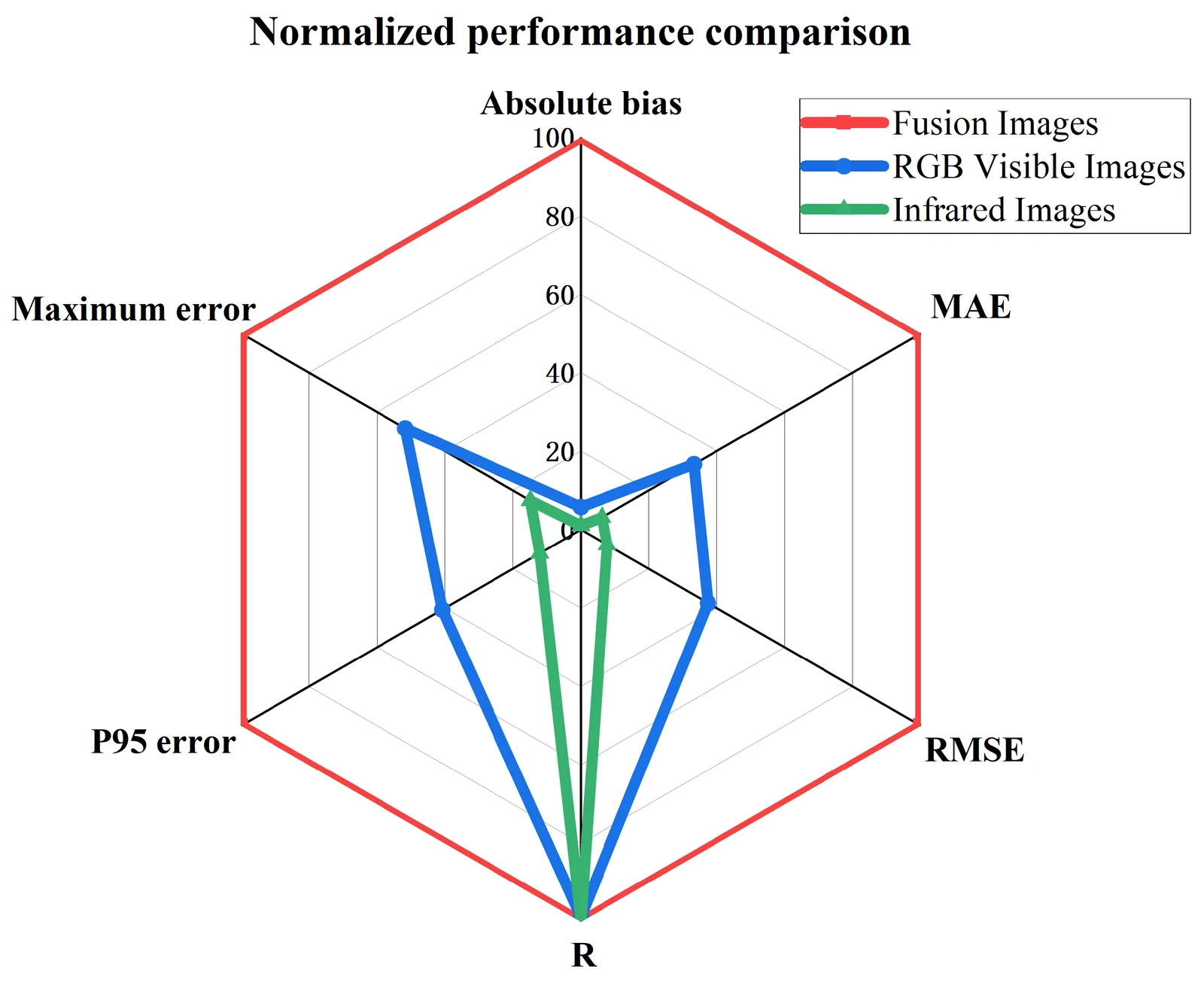
Normalized performance comparison of different image data sources. All metrics were converted to normalized performance scores from 0 to 100, with higher scores indicating better performance.

The effect of input modality was evaluated using visible images, infrared images, and fused images under the same data split and keypoint-evaluation procedure. Results are shown in Figure 12 and Figure 13 and Table 6. The fused images yielded the lowest absolute-error metrics and the smallest absolute bias. At the chicken level, the absolute errors obtained from fused images were significantly lower than those obtained from visible-only and infrared-only inputs (Holm-adjusted p=0.0059 and p=0.0039, respectively).

The distribution obtained from fused images was closest to the manual-measurement distribution, with both concentrated mainly between 80 and 90 mm. Fused images also achieved the best absolute bias, MAE, RMSE, P95 error, and maximum error. Visible images produced intermediate errors and a lower correlation. Infrared-only predictions retained a high R but were shifted toward lower values, producing a bias of −12.682 mm and an MAE of 12.682 mm. Their lower SD, CV, and $f_{e}$ indicated lower within-chicken variability across repeated acquisitions, but this did not correspond to greater measurement accuracy.

These results indicate that combining visible texture with infrared boundary cues improved endpoint-based shank length measurement relative to either input modality alone.

### 4.2. Ablation Study of the Fusion Model

**Figure 14:**
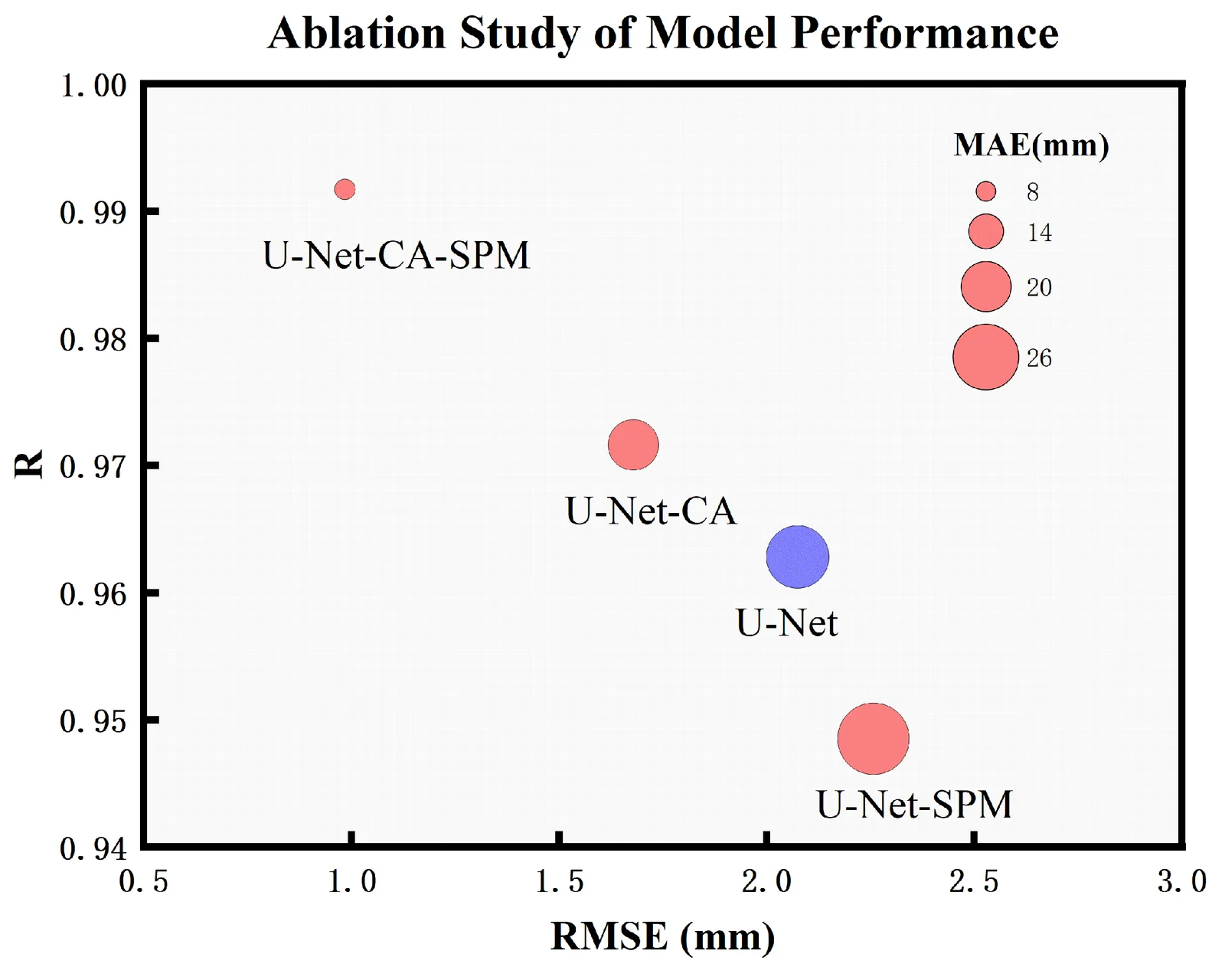
Bubble plot of the fusion model ablation study. The bubble size was scaled according to MAE, with larger bubbles indicating higher MAE. The numerical labels in the bubble-size legend indicate only the plotting scale used to display relative bubble sizes and do not represent MAE values in millimeters. The actual MAE values are reported in Table 7. The bubble color represents Bias (mm), changing from red to blue as Bias increases.

An ablation study used the same dataset and downstream keypoint-measurement procedure to isolate the effects of Coordinate Attention and Strip Pooling. Results are shown in Figure 14 and Table 7. U-Net-CA-SPM achieved the lowest MAE (0.790 mm), RMSE (0.984 mm), P95 error (1.751 mm), and maximum error (2.249 mm), together with the highest R (0.992). The bubble plot shows the same ranking for these metrics. Under the tested configuration, combining both modules therefore yielded the lowest downstream shank-length measurement errors.

The baseline U-Net produced an MAE of 1.712 mm, an RMSE of 2.074 mm, an R of 0.963, and the largest absolute bias among the four variants (0.858 mm). These results establish that the two attention-related additions improved downstream errors, but they do not by themselves identify the internal mechanism responsible for that improvement.

When introduced separately, CA and SPM produced different error profiles. U-Net-CA achieved a lower MAE and RMSE than U-Net-SPM and retained a higher R. This result is consistent with CA helping preserve spatially informative features, although feature-level analyses would be needed to confirm that interpretation. U-Net-SPM produced lower Bias, SD, CV, and $f_{e}$ but larger absolute-error metrics, showing that lower repeated-acquisition variability did not necessarily correspond to greater measurement accuracy. The combined model performed best, suggesting that the two modules provided complementary benefits under the tested conditions.

### 4.3. Comparison of Loss Functions for the Fusion Model

**Figure 15:**
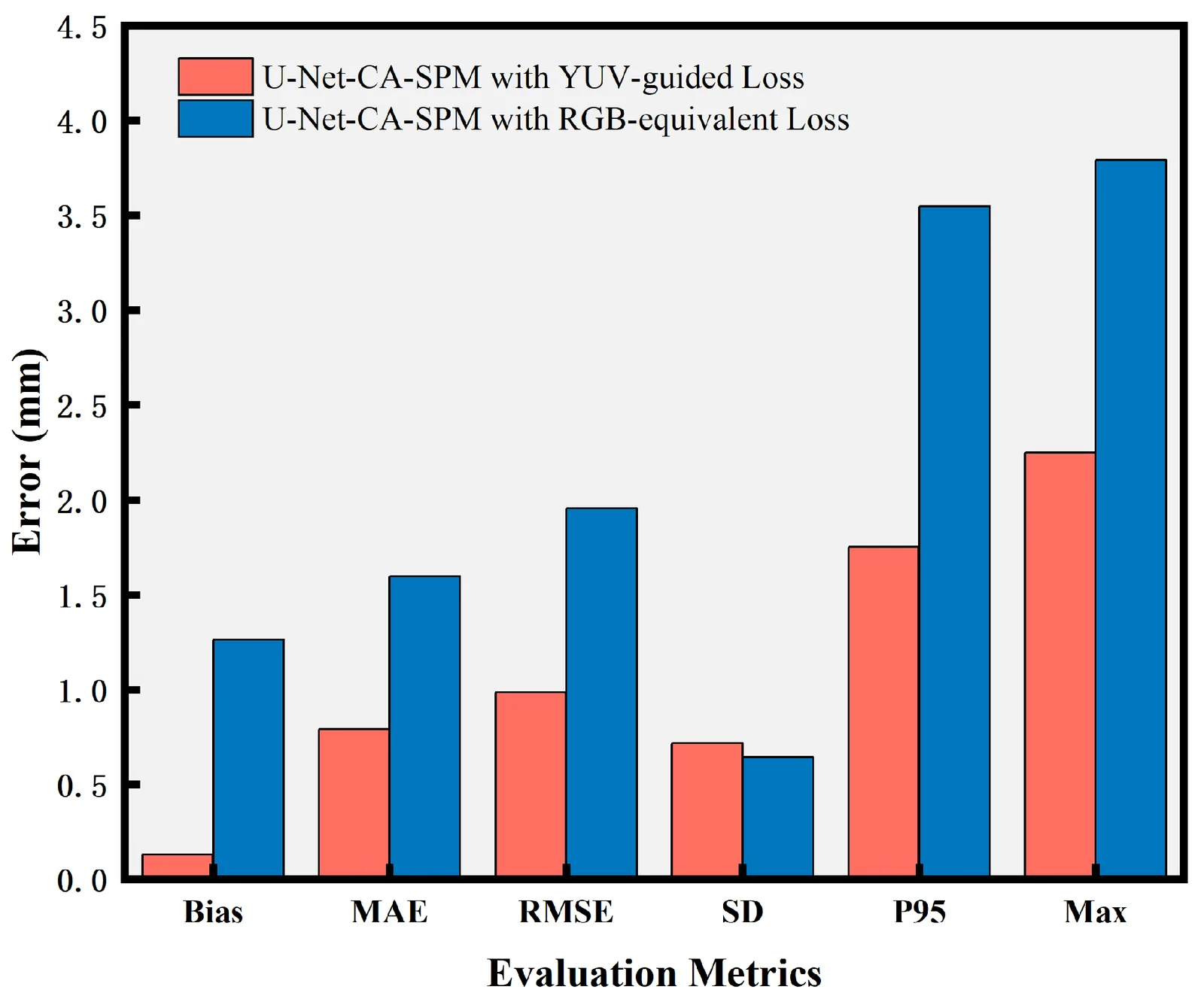
Error metric comparison of U-Net-CA-SPM with different loss functions.

YUV-guided and RGB-equivalent losses were compared using the same network architecture. Results are shown in Figure 15 and Table 8. YUV-guided loss reduced absolute bias, MAE, RMSE, P95 error, and maximum error, indicating closer agreement with manual measurements.

The main difference between the two formulations is that the YUV-guided loss applies separate pixel- and gradient-based constraints to luminance and chrominance components rather than constraining them in the RGB domain. The ablation results therefore support the empirical advantage of this differentiated supervision under the tested network architecture but do not establish which image regions or internal features were responsible for the improvement.

Together, the architecture and loss ablations show that U-Net-CA-SPM with YUV-guided loss produced the best downstream shank-length measurement performance among the tested fusion configurations.

### 4.4. Comparison of Different Image Fusion Methods

**Figure 16:**
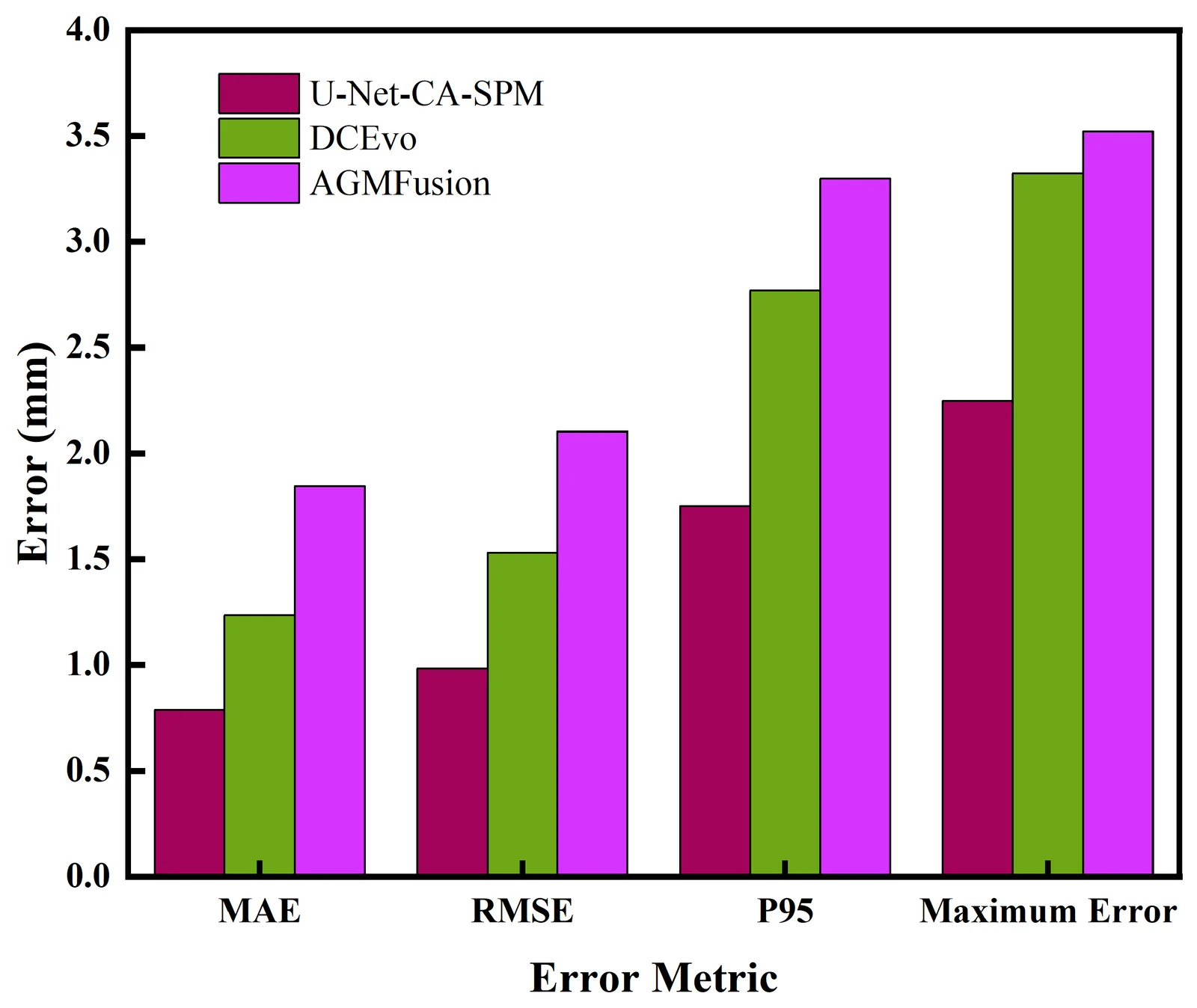
Comparison of shank length measurement errors among different image fusion methods.

To further compare the effects of different image fusion methods on shank length measurement, DCEvo and AGMFusion were compared with the proposed U-Net-CA-SPM. The three methods used the same individual-level data split, keypoint prediction model, training parameters, and chicken-level evaluation procedure. The results are shown in Figure 16 and Table 9. U-Net-CA-SPM achieved the smallest absolute Bias and lower MAE, RMSE, P95 error, and maximum error than DCEvo and AGMFusion, indicating lower overall measurement error, less systematic bias, and better control of large errors. Notably, AGMFusion achieved a higher R than DCEvo, whereas most of its measurement-error metrics were larger. This result shows that a higher correlation did not necessarily correspond to closer measurement agreement and that Bias and absolute-error metrics should therefore be considered together when evaluating the measurement performance of different fusion methods.

In terms of within-chicken repeatability, AGMFusion showed lower SD, CV, and $f_{e}$ than U-Net-CA-SPM, indicating that the different fusion methods exhibited different performance characteristics in measurement accuracy and repeatability. Because the fused images were used directly for subsequent TKP and BKP prediction, differences among the fusion methods were also reflected in the resulting keypoint prediction and shank length calculation. Overall, although U-Net-CA-SPM did not achieve the lowest values for all repeatability metrics, it showed smaller measurement errors, less systematic bias, and lower P95 and maximum errors. Under the dataset and experimental conditions used in this study, these results indicate that U-Net-CA-SPM was more suitable for the shank length measurement task investigated here.

### 4.5. Ablation Study of the Keypoint Prediction Model

**Figure 17:**
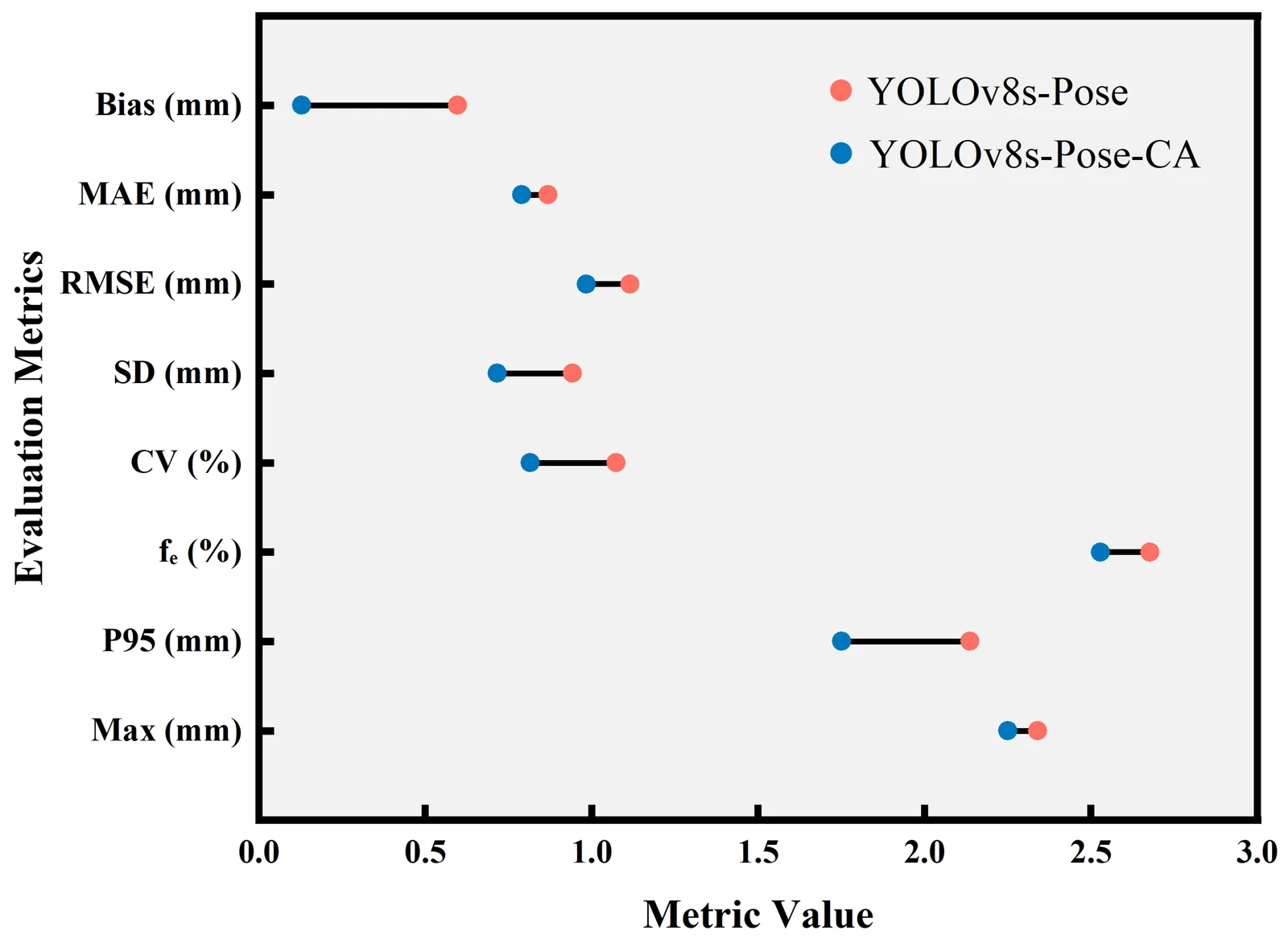
Effect of Coordinate Attention on shank length measurement performance.

The contribution of Coordinate Attention to the keypoint stage was evaluated by comparing YOLOv8s-Pose-CA with the original YOLOv8s-Pose. Both models used the same fused-image input, individual-level data split, and evaluation procedure. Results are reported in Table 10 and Figure 17.

As shown in Table 10, R changed only slightly after CA was introduced (0.991 to 0.992), whereas Bias, MAE, RMSE, P95 error, and maximum error all decreased. The dot-and-line plot in Figure 17 shows the same direction of change. Thus, CA primarily improved endpoint-derived error metrics rather than the overall correlation.

### 4.6. Ablation and Weight Sensitivity Analysis of the Length-Related Loss

In addition to the architectural modification, the effect of the length-related constraint on shank length measurement was further examined using four loss configurations. The original keypoint detection loss was compared with configurations using the MAE length term alone, the RMSE length term alone, and both terms together. The corresponding results are reported in Table 11.

The ablation results showed that using either length term alone did not consistently reduce the measurement errors. The MAE-only configuration showed lower variability-related metrics and smaller P95 and maximum errors than the original loss, but its MAE and RMSE remained slightly higher. The RMSE-only configuration also did not provide a consistent improvement. In contrast, combining the two length-related terms reduced the MAE from 1.483 to 0.790 mm and the RMSE from 1.858 to 0.984 mm, while the absolute Bias and relatively large errors were also reduced. This pattern is consistent with the different forms of the two constraints. $L_{len,MAE}$ directly penalizes the absolute deviation between the predicted and annotated shank segment lengths, whereas $L_{len,RMSE}$ gives greater influence to larger length deviations through the squared-error term. Under the tested settings, their combined use therefore provided more effective supervision of shank-length consistency than either term alone.

**Figure 18:**
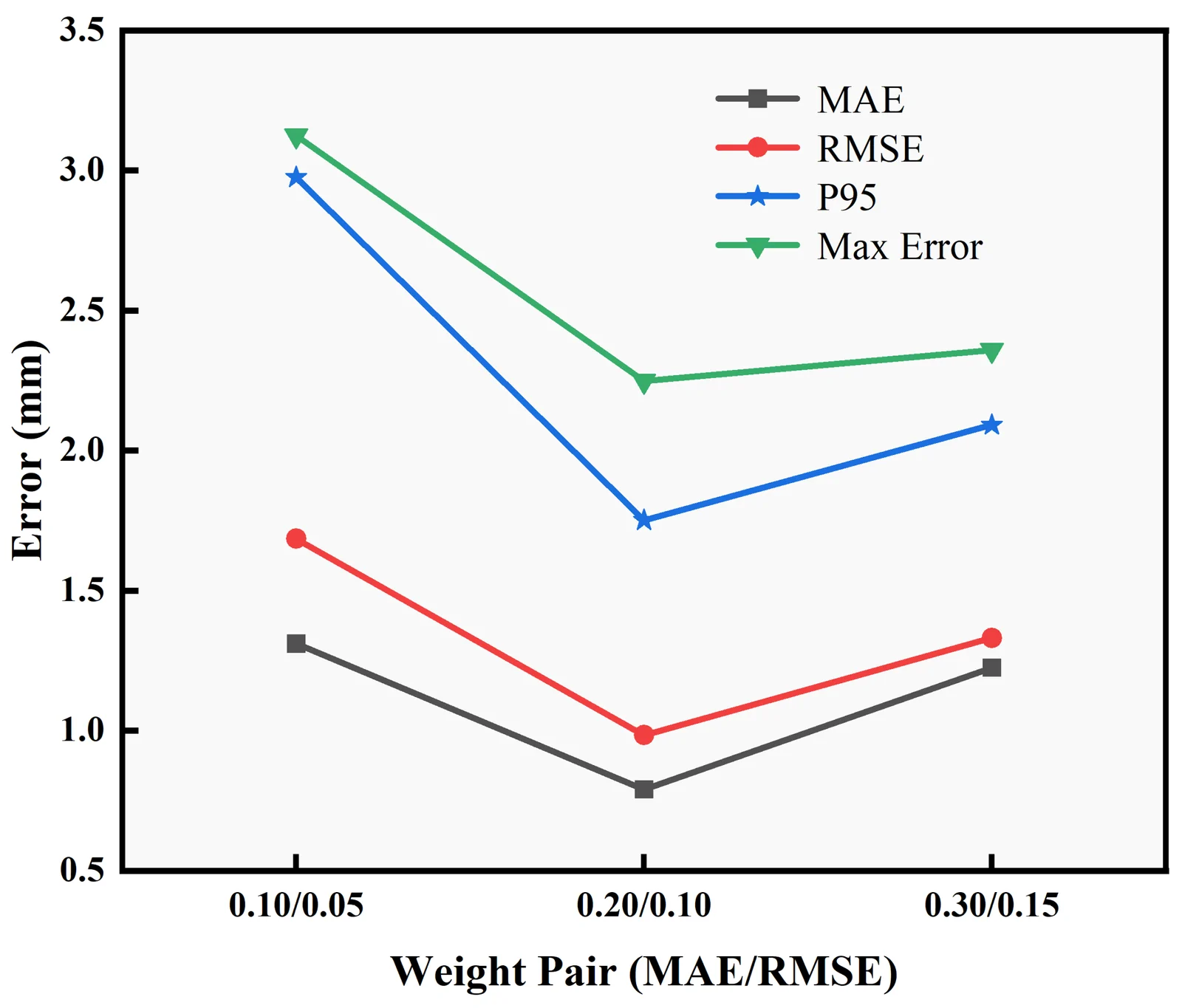
Weight sensitivity analysis of the length-related loss for shank length measurement.

The sensitivity of the combined loss to the weighting coefficients was further evaluated using three MAE/RMSE weight pairs: 0.10/0.05, 0.20/0.10, and 0.30/0.15. The results are summarized in Table 12, and the trends in the main error metrics are shown in Figure 18. The intermediate setting of 0.20/0.10 achieved the lowest MAE and RMSE, together with the smallest absolute Bias, P95 error, and maximum error among the three tested settings. Increasing the weights to 0.30/0.15 did not further improve the measurement accuracy, while reducing them to 0.10/0.05 resulted in larger absolute-error metrics.

The lower-weight setting produced slightly lower SD and CV than 0.20/0.10, while both settings had the same $f_{e}$ of 2.528%. Thus, the selected weights did not give the lowest value for every variability-related metric. However, 0.20/0.10 provided substantially lower measurement errors while maintaining similar variability. Because the two length-related terms are added to $L_{YOLOpose}$, changing their coefficients changes their relative contribution to the overall training objective. Among the tested settings, 0.20 and 0.10 provided the most favorable overall measurement performance and were therefore used in the final loss function.

### 4.7. Comparison of Keypoint Prediction Models

**Figure 19:**
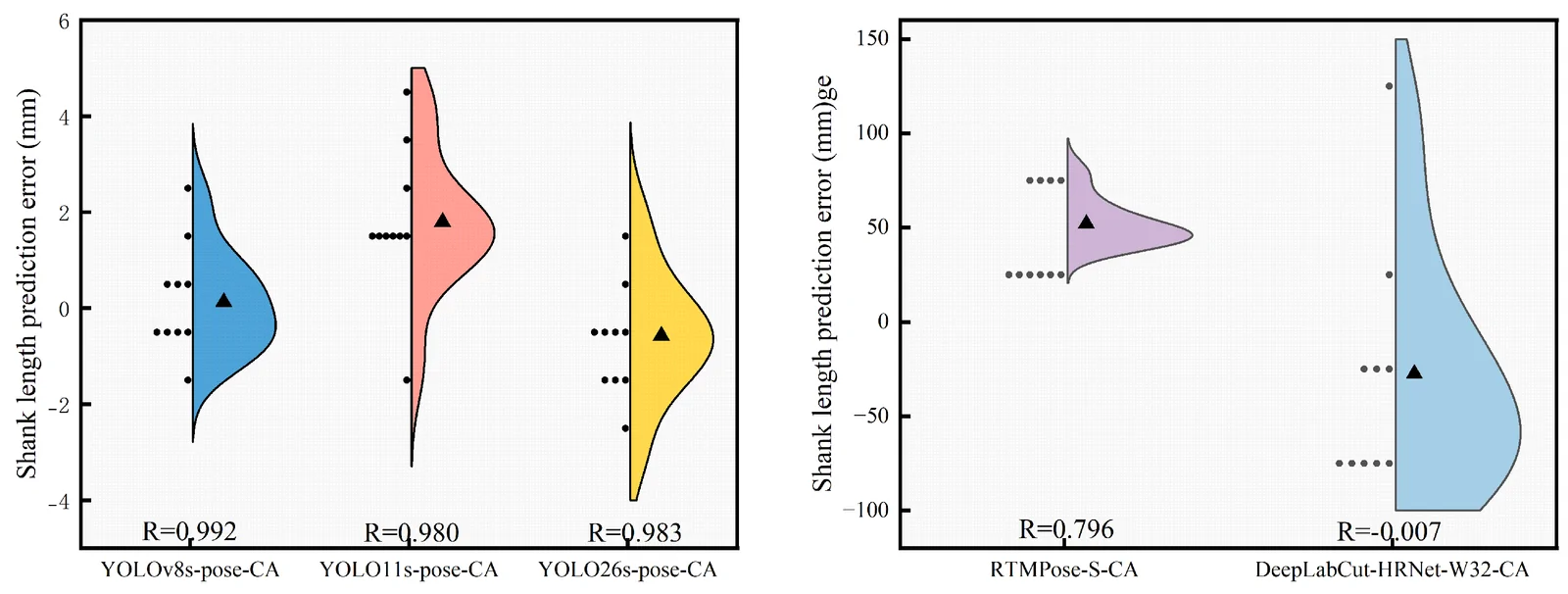
Distribution of shank length prediction errors for different keypoint prediction models. Prediction error was calculated as the difference between the shank length converted from predicted keypoints and the manually measured shank length. The colored violin shapes represent the distributions of prediction errors for the different models, the gray dots represent the individual chicken-level prediction errors, and the black triangles indicate the mean signed prediction error (Bias) for each model.

Five tested implementations were compared using the same fused-image input, individual-level split, and measurement procedure: YOLOv8s-Pose-CA, YOLO11s-Pose-CA, YOLO26s-Pose-CA, RTMPose-S-CA, and DeepLabCut HRNet-W32-CA. Each model predicted TKP and BKP, and the endpoint distance was converted to shank length. Results are reported in Figure 19 and Table 13.

As shown in Table 13, YOLOv8s-Pose-CA achieved the best performance for the main accuracy metrics. Its MAE and RMSE were 0.790 mm and 0.984 mm, respectively, and its R reached 0.992. Figure 19 further shows that the prediction errors of this model were mainly concentrated around 0 and had a relatively narrow distribution. This indicates that the model not only preserved the variation trend of chicken shank length effectively but also produced small measurement errors. Its shank-length estimates therefore showed high measurement accuracy and repeated-acquisition stability.

Compared with YOLOv8s-Pose-CA, YOLO26s-Pose-CA performed slightly worse overall in terms of the error metrics. Although YOLO26s-Pose-CA had lower SD and CV, indicating lower within-chicken variability across repeated acquisitions, its error distribution shifted slightly toward negative values. Its P95 error and maximum error were also higher than those of YOLOv8s-Pose-CA. This shows that lower variation did not translate into higher measurement accuracy. For the chicken shank length measurement problem studied in this work, improving measurement accuracy is more important while maintaining a relatively high level of repeatability.

The R value for YOLO11s-Pose-CA reached 0.980, indicating that its predictions followed a variation trend similar to that of the manually measured values. However, the half-violin plot shows that its prediction errors were generally positive, indicating a tendency toward overestimation. Its MAE and RMSE were both higher than those of YOLOv8s-Pose-CA, showing that its prediction accuracy was lower. Its SD, CV, and $f_{e}$ were also higher than those of YOLOv8s-Pose-CA, indicating greater within-chicken variability across repeated acquisitions. Therefore, although YOLO11s-Pose-CA maintained a relatively high correlation, it showed lower shank-length measurement accuracy and greater repeated-acquisition variability than YOLOv8s-Pose-CA.

The shank length values calculated from the keypoint predictions of RTMPose-S-CA and DeepLabCut HRNet-W32-CA differed greatly from the manually observed values. The half-violin plot shows that the prediction errors of RTMPose-S-CA were generally much greater than 0. Its Bias and MAE were both 51.980 mm, indicating that the predicted distance between TKP and BKP was generally too large and resulted in severe overestimation of the converted shank lengths. The error distribution of DeepLabCut HRNet-W32-CA covered a wide range and included large positive and negative deviations. This indicates that its predictions varied greatly and that the resulting shank-length estimates were not consistent. Its R value in Table 13 was only −0.007, while its MAE and RMSE reached 58.028 mm and 65.817 mm, respectively. These results also indicate that no clear linear relationship was observed between the predicted and manually measured shank lengths. Overall, under the experimental conditions of this study, neither model produced sufficiently consistent and accurate shank-length estimates from the predicted TKP and BKP. Therefore, they were not suitable as the preferred keypoint prediction model in the proposed shank length measurement procedure.

### 4.8. Model Complexity and Deployment Analysis

To further evaluate the deployment feasibility of the proposed method, the model complexity and inference efficiency of the final image fusion model and keypoint prediction model were analyzed. The tests were conducted on a single NVIDIA GeForce RTX 4090 D GPU with 24 GB of GPU memory. The experimental environment consisted of PyTorch 2.6.0+cu124 and CUDA 12.4. During testing, the batch size was set to 1. Each model was first warmed up for five runs, followed by 30 repeated inference runs to calculate the mean inference time. The inference time reported in this study included only the forward propagation process of each model. Image loading, format conversion, post-processing, and shank length conversion were not included. The results are shown in Table 14.

As shown in Table 14, most of the inference time of the proposed method was spent in the image fusion stage. The U-Net-CA-SPM image fusion model had only 1.670 M parameters and a weight size of 6.73 MB. However, because it performs pixel-level feature fusion and reconstruction of visible and infrared images, its mean forward propagation time for a single image was 42.56 ms. In comparison, the YOLOv8s-Pose-CA keypoint prediction model had more parameters, with 11.458 M parameters and a weight size of 69.34 MB, but its mean forward propagation time for a single image was 15.21 ms. This indicates that the model had relatively high inference efficiency during keypoint prediction. When the forward propagation processes of the image fusion and keypoint prediction models were performed sequentially, the total forward propagation time of the two-stage model was approximately 57.77 ms, corresponding to a processing speed of approximately 17.31 FPS. Overall, while using multi-source image fusion to improve measurement accuracy, the proposed method maintained an acceptable model size and inference efficiency, showing some feasibility for practical deployment.

### 4.9. Reference Comparison with Existing Shank Length Measurement Methods

For reference, the results reported in this study are presented alongside those of existing chicken shank length measurement methods in Table 15. Zheng et al. [1] reported SLCM, a live-chicken shank length measurement method based on regional keypoint extraction from visible images, whereas Ma et al. [12] reported SLM, which used fused visible and infrared images with an improved ResNet for direct shank-length regression. The reported results show that all three methods achieved relatively high correlations between vision-based and manual measurements, while their reported SD and CV values differed. However, these values were obtained using different datasets, acquisition systems, and experimental conditions. Therefore, the numerical results in Table 15 should be regarded as a reference comparison rather than a strict cross-study performance ranking. In addition, the present study reports Bias, MAE, and RMSE to characterize systematic and absolute measurement errors, which were not jointly reported by the two previous studies.

### 4.10. Limitations and Future Work

Several aspects of this study require further validation and improvement. The current dataset remains limited in the number of individuals and in the range of breeds and ages represented, and the generalizability of the model to broader chicken populations requires further validation. The method was also evaluated mainly under relatively stable acquisition conditions. In commercial poultry houses, illumination variation, motion blur, bird occlusion, and changes in camera-to-bird distance may affect image quality and keypoint localization. In addition, image acquisition currently requires the chickens to be caught and restrained. Although the vision-based measurement itself does not require direct contact with the shank, the overall acquisition procedure still depends on a certain degree of manual restraint. Future work will expand the sample size and individual diversity and conduct individual-level cross-validation and independent validation on larger datasets. The method will also be evaluated and optimized under more complex housing conditions and under low-restraint or freely moving conditions, with reduced reliance on restraint and manual handling. The potential effects of restraint on chicken stress and animal welfare will also be evaluated.

## 5. Conclusions

This study developed a chicken shank length measurement method based on visible and infrared image fusion and keypoint prediction for vision-based measurement without direct contact with the shank. First, the visible and infrared images were registered and fused to enhance the representation of edge, texture, and thermal information in the chicken shank region. The fusion images were then used as input to the improved YOLOv8s-Pose-CA model to predict the two keypoints required for shank length measurement, namely TKP and BKP. Finally, the distance between the two points was converted into the corresponding shank length.

The experimental results showed that the shank length measurements obtained from the fusion images were better than those obtained from either visible or infrared images alone. This indicates that multi-source image information can provide more effective input for subsequent keypoint prediction. The U-Net-CA-SPM fusion model combined with YUV-guided loss further reduced the measurement errors. This demonstrates that the introduced Coordinate Attention module, Strip Pooling module, and loss function design had positive effects on fusion image quality and subsequent shank length measurement. In the final test, the Bias, MAE, and RMSE of the proposed method were 0.129 mm, 0.790 mm, and 0.984 mm, respectively, and the R value between the predictions and the manual measurements reached 0.992. The comparison of the keypoint prediction models also showed that YOLOv8s-Pose-CA performed better than the other models, including YOLO11s-Pose-CA, in terms of overall error, distribution agreement, and extreme error control. It produced more accurate shank-length estimates based on the predicted keypoints.

In summary, the proposed method can measure the shank length of live chickens with relatively high accuracy and provides a feasible approach for the automated vision-based acquisition of poultry body size parameters without direct contact with the measured body region.

## Figures and Tables

**Table 1:** Affine transformation parameters used to register the visible and infrared images.

Parameter	Value
$s_{x}$	1.100
$s_{y}$	1.182
θ	$-0.8^{\circ }$
$t_{x}$	−173 px
$t_{y}$	−319 px

**Table 2:** Training hyperparameters of the main models.

Experiment Type	Input Size	Batch Size	Max Epoch	Optimizer
Image fusion ablation, loss comparison, and fusion-method comparison	1088 × 816	8	50	Adam
YOLO-based keypoint prediction comparison, loss ablation, and weight sensitivity analysis	1024 × 1024	8	180	Ultralytics auto
RTMPose-S-CA framework comparison	1024 × 1024	4	200	AdamW
DeepLabCut HRNet-W32-CA framework comparison	1024 × 1024	4	200	AdamW

**Table 3:** Endpoint localization errors of TKP and BKP on the 100 fused test images.

Endpoint	Mean Error (px)	Median Error (px)	P95 Error (px)	Mean Normalized Error (%)
TKP	8.373	8.236	13.958	1.354
BKP	10.566	8.480	27.384	1.691

**Table 4:** Evaluation results of the proposed shank length measurement method.

Metric	Value
MAE (mm)	0.790
RMSE (mm)	0.984
R	0.992
Bias (mm)	0.129
P95 (mm)	1.751
Maximum error (mm)	2.249

**Table 5:** Within-chicken repeatability of model-derived shank lengths across ten repeated acquisitions per chicken.

Metric	Mean	Range
Within-chicken SD (mm)	0.716	0.466–1.085
Within-chicken CV (%)	0.815	0.532–1.056
Within-chicken $f_{e}$ (%)	2.528	1.652–3.242

**Table 6:** Performance comparison of shank length measurement using different image data sources.

Metric	Fusion Images	Visible Images	Infrared Images
Bias (mm)	0.129	−2.229	−12.682
MAE (mm)	0.790	2.374	12.682
RMSE (mm)	0.984	2.631	12.745
R	0.992	0.980	0.986
SD (mm)	0.716	0.784	0.556
CV (%)	0.815	0.963	0.740
$f_{e}$ (%)	2.528	2.994	2.329
P95 (mm)	1.751	4.291	14.501
Maximum error (mm)	2.249	4.352	15.156

**Table 7:** Ablation results of the image fusion model for shank length measurement.

Metric	U-Net-CA-SPM	U-Net-SPM	U-Net-CA	U-Net
Bias (mm)	0.129	0.052	0.120	0.858
MAE (mm)	0.790	1.911	1.454	1.712
RMSE (mm)	0.984	2.257	1.678	2.074
R	0.992	0.949	0.972	0.963
SD (mm)	0.716	0.603	0.784	0.756
CV (%)	0.815	0.683	0.856	0.846
$f_{e}$ (%)	2.528	2.263	2.747	2.984
P95 (mm)	1.751	3.975	2.643	3.599
Maximum error (mm)	2.249	5.055	2.938	4.891

**Table 8:** Comparison of measurement performance with different loss functions in U-Net-CA-SPM.

Metric	U-Net-CA-SPM with YUV-Guided Loss	U-Net-CA-SPM with RGB-Equivalent Loss
Bias (mm)	0.129	1.263
MAE (mm)	0.790	1.597
RMSE (mm)	0.984	1.955
R	0.992	0.980
SD (mm)	0.716	0.644
CV (%)	0.815	0.708
$f_{e}$ (%)	2.528	2.273
P95 (mm)	1.751	3.548
Maximum error (mm)	2.249	3.792

**Table 9:** Comparison of shank length measurement performance using different image fusion methods.

Metric	U-Net-CA-SPM	DCEvo	AGMFusion
Bias (mm)	0.129	−1.052	−1.846
MAE (mm)	0.790	1.236	1.846
RMSE (mm)	0.984	1.532	2.104
R	0.992	0.987	0.989
SD (mm)	0.716	1.012	0.594
CV (%)	0.815	1.165	0.674
$f_{e}$ (%)	2.528	3.849	2.209
P95 (mm)	1.751	2.771	3.298
Maximum error (mm)	2.249	3.324	3.521

**Table 10:** Effect of Coordinate Attention on YOLOv8s-Pose for shank length measurement.

Metric	YOLOv8s-Pose-CA	YOLOv8s-Pose
Bias (mm)	0.129	0.596
MAE (mm)	0.790	0.869
RMSE (mm)	0.984	1.115
R	0.992	0.991
SD (mm)	0.716	0.942
CV (%)	0.815	1.074
$f_{e}$ (%)	2.528	2.677
P95 (mm)	1.751	2.136
Maximum error (mm)	2.249	2.340

**Table 11:** Ablation results of the length-related loss for shank length measurement.

Metric	Original Loss	MAE-Only Loss	RMSE-Only Loss	Combined Loss
MAE weight	0	0.20	0	0.20
RMSE weight	0	0	0.10	0.10
Bias (mm)	0.509	1.306	0.931	0.129
MAE (mm)	1.483	1.610	1.715	0.790
RMSE (mm)	1.858	1.876	2.054	0.984
R	0.970	0.982	0.966	0.992
SD (mm)	0.887	0.731	0.897	0.716
CV (%)	0.970	0.787	1.003	0.815
$f_{e}$ (%)	3.087	2.619	3.248	2.528
P95 (mm)	3.294	3.135	3.432	1.751
Maximum error (mm)	3.596	3.435	3.461	2.249

**Table 12:** Sensitivity of shank length measurement performance to the weights of the length-related loss terms.

Metric	0.10/0.05	0.20/0.10	0.30/0.15
MAE weight	0.10	0.20	0.30
RMSE weight	0.05	0.10	0.15
Bias (mm)	1.248	0.129	0.272
MAE (mm)	1.311	0.790	1.225
RMSE (mm)	1.685	0.984	1.331
R	0.988	0.992	0.983
SD (mm)	0.712	0.716	0.843
CV (%)	0.780	0.815	0.967
$f_{e}$ (%)	2.528	2.528	3.205
P95 (mm)	2.975	1.751	2.092
Maximum error (mm)	3.125	2.249	2.359

**Table 13:** Comparison of keypoint prediction models for shank length measurement.

Metric	YOLOv8s-Pose- CA	YOLO11s-Pose- CA	YOLO26s-Pose- CA	RTMPose-S-CA	DeepLabCut HRNet-W32-CA
Bias (mm)	0.129	1.797	−0.571	51.980	−27.517
MAE (mm)	0.790	2.019	1.099	51.980	58.028
RMSE (mm)	0.984	2.280	1.387	53.242	65.817
R	0.992	0.980	0.983	0.796	−0.007
SD (mm)	0.716	0.857	0.532	5.088	75.058
CV (%)	0.815	0.955	0.601	3.502	103.597
$f_{e}$ (%)	2.528	3.295	2.048	12.184	713.344
P95 (mm)	1.751	4.079	2.483	72.878	99.374
Maximum error (mm)	2.249	4.303	2.944	79.234	108.497

**Table 14:** Model complexity and inference efficiency of the proposed method.

Module	Input Size	Parameters/M	Weight Size/MB	Average Inference Time/ms	FPS
U-Net-CA-SPM	1088×816	1.670	6.73	42.56	23.49
YOLOv8s-Pose-CA	1024×1024	11.458	69.34	15.21	65.76
Overall measurement pipeline	–	13.128	76.07	57.77	17.31

**Table 15:** Reference comparison of reported results from different chicken shank length measurement studies.

Method	R	SD (mm)	CV (%)
SLCM	0.8722	1.35	2.00
SLM	0.996	0.181	0.21
Ours	0.992	0.716	0.815

## Data Availability

The data presented in this study are available on request from the corresponding author.
